# Supplementary material for: Modularity and composite diversity affect the collective gathering of information online
Source: Nat Commun. 2021 May 27;12:3195. doi: 10.1038/s41467-021-23424-1 (PMC8159948; doi:10.1038/s41467-021-23424-1)
Supplement: Supplementary file 1 — Supplementary Information [file 41467_2021_23424_MOESM1_ESM.pdf]

Modularity and Composite Diversity Affect the Collective Gathering of Information Online

Niccolò Pescetelli*, Alex Rutherford, Iyad Rahwan[1]

*Corresponding Author: Niccolò Pescetelli., E-mail: niccolo.pescetelli@gmail.com

1 Supplementary Methods

Procedure. The experimental procedure and main analyses were pre-registered at AsPredicted.org. Code to reproduce figures and analysis as well as data files are available via OSF (https://osf.io/wb538). Participants (N=193) were recruited through Amazon Mechanical Turk. The experiment was divided into a pre-test and a test phase. Three days before the actual experiment (pre-test phase), participants answered a battery of demographic, cognitive and personality questionnaires. Survey answers were pre-processed and used to assign participants to each experimental condition. Two factors were manipulated, namely group diversity (Low vs. High) and crowd modularity (Lo vs. Hi). Notice that we use a different spelling for Modularity to distinguish it from Diversity. Depending on their assigned condition, participants received an email with their specific login details. On the day of the experiment (test phase), participants were asked to login on a custom web application that was developed for this study and is available upon request. The main experiment lasted approximately 1 hour. Participants gave their informed consent. The study was approved by the MIT IRB board.

Pre-test phase. At pre-test, demographic, relational, cognitive and personality measures were collected. Questions selected represented a subsample of questions selected by the authors in collaboration with the MATRICS team in the IARPA's Hybrid Forecasting Challenge. They were selected to measure a comprehensive list of variables, often associated with diversity in the literature and explained here. Notice that the goal in selecting a heterogeneous metrics is to capture the intuition behind the use of high-dimensional user profiling and segmentation online. Thus, instead of drawing conclusions on a specific aspect of diversity, our aim is to see whether diversity broadly defined ('trait' diversity) might be reflected in informational diversity when individuals use online tools to retrieve task-relevant information ('state'). Survey answers were used to place each participant along a D-dimensional space ($D = 29$), represented by the vector Θ, where each dimension had an equal weight. Demographic measures included 26 questions, each contributing as one dimension of the space Θ. All measures were treated as a continuous scale. They spanned across multiple areas, including: (1) personal information and demographics (e.g., age, gender, sexual orientation, ethnicity, years of education), often associated with demographic or 'surface' diversity [1, 2]; (2) work & skills (e.g., previous and current job, daily hours spent on different skills, etc.), often associated with functional diversity [3, 1]; (3) geography and politics (e.g., number of countries lived, languages spoken, political orientation), also associated with opinion and 'deep-level' diversity [2]; (4) relational indicators (e.g., number of languages spoken by the average peer), recently associated with group or network diversity [4]. A complete list is reported below. Cognitive and personality measures were also included by the MATRICS team to assess to various degrees system 2 and reflective thinking abilities. The following were included in this study, as associated with diversity in mental models and reasoning styles [5, 6, 2]: (1) Cognitive Reflective Test (CRT) [7]; (2) the Active Open-Mindedness Test (AOMT) [8]; (3) and the Need For Cognition test (NFC) [9]. The global score to each these tests provided three further dimensions on Θ. Survey questions were primarily chosen from a larger pool of intake measures that were administered to volunteers in the IARPA Hybrid Forecasting Competition (HFC). All measures and tests were chosen because of their similarity with the HFC intake survey and their ease of use in an online experimental context. We report the distribution of responses for each population segment (Core, Inner, Outer) as defined below (Supplementary Figures 20-21).

Experimental manipulation. Two factors expected to affect group performance were orthogonally manipulated: Diversity and Modularity. One of the challenges of this experimental design was to randomize participants into groups that differed in diversity, defined by group members' distance along the Θ space. Given that any demographic, cognitive or personality measure cannot by definition be randomized, diversity is typically observed rather than experimentally manipulated. Our solution consisted of stratifying our volunteers population into segments that differed based on their distance from the center mass of the population's distribution and then

proceeded to assign participants close to the center (test participants) to either a close-by segment or a distant segment of the distribution's tail (treatment participants) (Figure 1 in the main text). To this end, all survey measures were first normalized across participants. The clustering algorithm DBSCAN was then applied to the normalized scores and used to divide participants into three groups: Core, Inner Periphery and Outer Periphery, corresponding to the algorithms' core points, (density-)reachable points and outliers [10]. These three sets of points represent the participant's proximity to the center of the distribution on the space Θ. Figure 1a in the main text represents this segmentation in a normal distribution along a one dimensional Θ. DBSCAN depends on two parameters, ϵ and μ, controlling the maximum distance allowed between within-cluster points and the minimum number of points within a cluster respectively. We selected ϵ and μ parameter values so that: (1) one single Core cluster was found; (2) the Core included approximately double the number of points assigned to the Inner Periphery and Outer Periphery segments; (3) Inner Periphery and Outer Periphery included about the same number of points. The chosen values were $\epsilon = 4.799999999999997$ and $\mu = 24$. Participants assigned to the Core should then cluster around the mean of the response distribution, while participants marked as (density-)reachable (Inner Periphery) or outliers (Outer Periphery) should lie in the tails of the distribution. By definition, Core participants are more similar to participants in the Inner Periphery rather than in the Outer Periphery. Two diversity conditions (Low vs. High) were thus created by randomly assigning participants in the Core to participants in the Inner Periphery (Low diversity) or in the Outer Periphery (High diversity). This manipulation uses a subsample of the population (Inner and Outer) as treatment for another subsample (Core). Participants in the Core segment can thus be randomized to interact with one of two treatments, corresponding to similar or different individuals. It allows to effectively measure the causal effect of group diversity on group performance, by comparing the effect that interacting with similar or dissimilar individuals has on Core (test) participants.

The second factor that was manipulated was Modularity. In particular, we asked the question: does a crowd of N individuals perform better as one single group ($M = 1$) or as multiple smaller and insulated groups ($M > 1$)? We randomly assigned participants in each diversity segment (Core, Inner, Outer) to two Modularity conditions (Lo vs. Hi). Participants in the Lo condition were put into one single group ($M = 1$) and discussed their views with everyone else in that condition. Participants in the Hi condition on the contrary were divided up into several groups ($M > 1$) and thus discussed their views with a smaller fraction of participants in their same condition (Figure 1b in the main text). A total of 193 participants who responded to the initial survey were thus divided into 14 groups, two of them assigned to the Lo condition (one High and one Low diversity) and twelve to the Hi condition (six High and six Low diversity) (see Supplementary Table 2).

Test phase. Three days after the initial survey, participants participated in the main experiment. They logged into a custom online platform built for this purpose using personal credentials emailed to them in advance. After giving their consent, participants answered 8 individual forecasting problems (IFP), randomly selected from a larger pool of binary forecasting problems released within IARPA's HFC and unresolved (i.e., whose solution was unknown) at the time of the experiment. It is important to stress the fact that these represent real complex problems that analysts and government agencies face every day and for which there is no trivial solution. For each IFP, participants went through three timed consecutive stages. To be consistent with Navajas et al.'s study [11], timing for question was calculated so to present 8 forecasting problems within the duration of the experiment (one hour). During stage one, participants saw a binary question regarding a real geopolitical event (all IFPs reported in Appendix) and had 30 seconds to enter an Initial private forecast off the top of their heads (*initial*). During stage two, they had 1½ minutes to search relevant information online and enter a revised private forecast (*revised*). Although short, the goal of stage 2 was to simply prime people with the first online information they could find in their digital sphere, rather than allowing a critical appraisal of the topic or the reading of in-depth articles on the subject. Finally, during the third and last stage (4½ minutes), participants discussed in real time their views using an inbuilt chat. During this stage, participants had to agree on a joint forecast (*consensus*) as well as giving their final private forecast (*final*). Consensus forecasts had to be the same for all members of a group for the answer to be valid and thus represented collective beliefs. Final forecasts on the contrary could differ across members and thus represented private beliefs after social interaction. Differences between the two would indicate differences in forecasting accuracy due to different elicitation mechanisms. Participants were rewarded for both time spent (max \$5) and accurate responding (max \$6.40). Each forecast could yield a maximum of \$0.20, inversely proportional to their Brier forecasting error (see §Task and Performance).

Task and Performance. All IFPs were randomly selected from a larger pool of binary IFPs posed within the IARPA Hybrid Forecasting Competition and unresolved at the time of the experiment. IARPA's IFPs are chosen to reflect realistic forecasting problems that analysts face in their daily activity. Our task thus represents a realistic and ecologically valid problem set. Like in the IARPA HFC tournament, performance was measured with a quadratic error measure called Brier score [12]. Brier scores represent a proper scoring

rule for probabilistic responses and a measure of second-order accuracy or *calibration* [13], which means that they penalize people both for being over- and under-confident. For a binary question, a brier score is computed according to Equation 1 in the main text.

Exclusion criteria. Our manipulation consisted of randomly matching participants in the Core stratum (test participants) with participants in the Inner or Outer periphery (treatment participants). Due to attrition rates however, the actual number of participants in each segment who came back for the test phase was not under our control. Random fluctuations in participants attendance were particularly consequential in smaller groups. We adopted a conservative cutoff, keeping for further analysis all groups in which at least one participant in each segment showed up. Adopting this criterion, two High-Hi groups were removed. The final number of participants (N) and groups (M) kept for further analysis is shown in Supplementary Table 3.

Manipulation checks. Fewer participants than initially recruited came back to the test phase (N=112, $\sim$ 41.9% dropout rate) and further were removed after applying our exclusion criteria defined above (final N=104). Supplementary Figure 1a and Supplementary Table 3 show the final groups tested in each condition and the test/treatment composition of each group. A t-test between the two diversity conditions showed that the drop-out rate was orthogonal to and thus did not affect our diversity manipulation ($t = 2.65, p = .02, d = 1.55$).

Analysis. Data analysis was performed in Python 3.5 and R 3.6. Analyses were performed on forecasting errors measured in Brier scores [12], and separately computed for forecasts made at different stages (initial, revised, consensus and final) of each IFP. All continuous predictors were standardized before being entered in the regression, all categorical variables were declared as factors. Errors were fitted with multi-level Generalized Linear Mixed-effects Models (GLMM) with a Gaussian log link function (R `glmer` in the `lme4` package, family=gaussian, link=log). The log transformation had the benefit of reducing the skewness usually observed in Brier score distributions (Supplementary Figure 2). The use of GLMM is to be preferred to the application of linear models to transformed data [14]. Furthermore, abundant evidence in psychology suggests a general cognitive mechanism for quantities estimation based on logarithm scales [15], which in turn produces log-scale variability in judgment errors [16]. Similar conclusions were reached using Gaussian family and logit link (Supplementary Table 17). However, although quantifying forecasting accuracy in Brier scores represents a standard [17], the same models fitted to Brier scores (second order accuracy) were also tested on binarized forecasts (first order accuracy) with the parameters (family=Binomial, link="Logit") (Supplementary Tables 11, 12, 13, 14). All analyses were limited only to participants who fell in the Core segment (i.e., test participants), as these were the only ones to whom the true randomization applied. This allows us to draw causal inferences on the effect of our manipulation, as all Core participants were equal in expectation. Analyses were conducted both at the individual and aggregate level (i.e., group-level). At the individual level, we compared individual forecasts made by participants across different conditions and stages. At the group-level, we aggregated individual forecasts in each condition with a median rule and compared how our experimental manipulation affected such aggregate forecast accuracy. In all following analyses and unless specified otherwise, we included a random intercept for each IFP, so to account for differences in IFP difficulty.

All communications among users happened through a built-in chat, recording all linguistic events. We analyzed linguistic interactions using a set of standard measures. These were: (i) Vocabulary size: the size of the set of words used in all messages by all participants for a given question. Words that appear very frequently in English (stop words) were excluded. (ii) Vocabulary entropy: the Shannon entropy of the frequency distribution of words in the vocabulary. A high entropy indicates words in the vocabulary are used more equally, while a lower entropy means a small number of words are used more frequently than the rest. (iii) Message size: the total number of messages exchanged by the participants. (iv) User entropy: the Shannon entropy of the frequency distribution of messages by users. A higher value indicates users participated more equally. (v) Messages per user: the mean number of messages per user; the total number of messages exchanged divided by the number of participants. Measures were computed for each group and each question separately.

2 Note on terminology

Notice that there is a subtle difference between 'modularity'—defined by the number of groups M in which N participants are divided into—and 'group size'—defined as N/M. Although our modularity factor exactly coincides with our group size condition (*i.e.*, participants in the high modularity condition are also in the small group condition, and vice versa), these two are conceptually distinct and can be investigated with different sets of analysis. In the main text, we try to use the two terms accordingly. Specifically, we use 'group size' for most of our analyses, given that this is the only variable that our participants experience. We use instead 'modularity' to describe our factorial manipulation and for all analyses that involve aggregating data across subgroups/modules (*e.g.*, in the case of our aggregated Brier measure).

3 Demographics variables

Below are the 26 demographic indicators used to define the space Θ. On top of these axes, three more dimensions were added based on the aggregate scores of the CRT, AOMT and NFC tests.

1. Age,
2. Sex
3. Where do you place yourself along the continuum from Completely Heterosexual (0) to Completely Homosexual (100)
4. Years of education
5. Thinking about your current or most recent job (includes unpaid roles, such as student or homemaker), specify how many hours a day you spend on each of the following types of activities. - People - Working to help, lead, or persuade people (e.g., teaching, sales, coaching)
6. Thinking about your current or most recent job (includes unpaid roles, such as student or homemaker), specify how many hours a day you spend on each of the following types of activities. - Things - Working with physical objects, machines, or materials (e.g., building, transporting, landscaping)
7. Thinking about your current or most recent job (includes unpaid roles, such as student or homemaker), specify how many hours a day you spend on each of the following types of activities. - Ideas - Working with abstract ideas, plans, or concepts (e.g., creating plans, artistic designs, theories, or stories)
8. Thinking about your current or most recent job (includes unpaid roles, such as student or homemaker), specify how many hours a day you spend on each of the following types of activities. - Data - Working with data or numbers (e.g., data analysis, budget planning, accounting)
9. Thinking about your previous job (includes unpaid roles, such as student or homemaker), specify how many hours a day you were spending on each of the following types of activities. - People - Working to help, lead, or persuade people (e.g., teaching, sales, coaching)
10. Thinking about your previous job (includes unpaid roles, such as student or homemaker), specify how many hours a day you were spending on each of the following types of activities. - Things - Working with physical objects, machines, or materials (e.g., building, transporting, landscaping)
11. Thinking about your previous job (includes unpaid roles, such as student or homemaker), specify how many hours a day you were spending on each of the following types of activities. - Ideas - Working with abstract ideas, plans, or concepts (e.g., creating plans, artistic designs, theories, or stories)
12. Thinking about your previous job (includes unpaid roles, such as student or homemaker), specify how many hours a day you were spending on each of the following types of activities. - Data - Working with data or numbers (e.g., data analysis, budget planning, accounting)
13. Race/EthnicityUse the sliders to describe your ethnicity or mixture thereof. - American Indian (Native American) or Alaska Native
14. Race/EthnicityUse the sliders to describe your ethnicity or mixture thereof. - Asian or Asian American
15. Race/EthnicityUse the sliders to describe your ethnicity or mixture thereof. - Black or African American
16. Race/EthnicityUse the sliders to describe your ethnicity or mixture thereof. - Hispanic or Latinx or Spanish Origin
17. Race/EthnicityUse the sliders to describe your ethnicity or mixture thereof. - Native Hawaiian or Other Pacific Islander
18. Race/EthnicityUse the sliders to describe your ethnicity or mixture thereof. - White
19. Race/EthnicityUse the sliders to describe your ethnicity or mixture thereof. - Other
20. How do you consider yourself? - West-East
21. What is your political orientation from 0 (Far left) to 100 (Far right)
22. What is the political orientation of your average friend from 0 (Far left) to 100 (Far right)

23. How many countries have you lived in for more than 6 months?
24. How many countries has your average friend lived in for more than 6 months?
25. How many languages do you fluently speak?
26. How many languages does your average friend fluently speak?

4 Accuracy-based analyses

All Generalized Linear Mixed-effects Models (GLMM) fitted on Brier scores were also fitted on binarized forecast accuracy (0: incorrect, 1:correct). While Brier scores represent a measure of calibration [13] - namely accuracy in the estimation of uncertainty (second-order accuracy) - binarized accuracy represents only a measure of accuracy of the most likely outcome (first-order accuracy) and it is therefore a less granular measure. We discretized the outcome variables by applying a criterion of 0.5 on the Brier scale - corresponding to a forecast of 50% - so that all Brier scores smaller than 0.5 were assigned a value of 1 (correct) and Brier scores greater than 0.5 were assigned a value of 0 (incorrect). The new GLMMs were run with family=binomial(link='Binomial') parameters. Results are reported in Supplementary Tables 11-14.

Together with the results reported in the main text, the findings show that, when using binarized accuracy rather than Brier scores as dependent variable, some similarities but also some differences emerged. At the individual level, we first replicate the difference existing between asocial (stage 1 and 2) and social (stage 3) forecasts but show that no difference in accuracy exists between social forecasts (final vs. consensus). Second, we replicate the interaction between the two experimental factors (Diversity X Modularity) but not the main effects of each factor. At the aggregate level, we find no difference in binarized accuracy between forecast types nor any effect of our experimental manipulation. Overall, the results suggest that our paradigm created measurable differences between first order (i.e., binarized accuracy) and second order (i.e., Brier scores) accuracy [13].

5 Linguistic analysis

All social interactions during the test phase happened through a built-in online chat that recorded all linguistic interactions between players in the same group. Measures considered were: (i) Vocabulary size: the size of the set of words used in all messages by all participants for a given question. Words that appear very frequently in English (stop words) were excluded. (ii) Vocabulary entropy: the Shannon entropy of the frequency distribution of words in the vocabulary. A high entropy indicates words in the vocabulary are used more equally, while a lower entropy means a small number of words are used more frequently than the rest. (iii) Message size: the total number of messages exchanged by the participants. (iv) User entropy: the Shannon entropy of the frequency distribution of messages by users. A higher value indicates users participated more equally. (v) Messages per user: the mean number of messages per user; the total number of messages exchanged divided by the number of participants. Measures were computed for each group and each question separately. Each linguistic measure's distribution and correlations between each pair of measures is plotted in Supplementary Figure 4 and 5. The figure shows also the correlation with Brier scores to test whether any correlation existed between linguistic indicators and forecasting accuracy. Results suggest that participants in diverse groups (a) use less unique words (b) send significantly less messages per user ($p = .02$) and (c) show a trend towards sending less messages overall. When dividing by modularity condition instead, we notice that larger groups (Lo Modularity) tend to send more messages and use more words overall. Although this is simply due to the presence of more people joining the conversation, larger groups also seems to show a marginal increase of message per participant. Finally, in larger groups words seem to be used more equally (meaning higher vocabulary entropy) and turn taking between participants is higher (meaning higher user entropy).

6 Consensus reaching

The ability to look into the chat data gave us access to another important source of information, namely the dynamics of opinion aggregation over time. To analyze how consensus was reached, we analyzed numerical estimates suggested in the chat over the course of the online conversation. We used an automated method to extract all numerical estimates that were mentioned during the chat conversations. We then checked by hand the data (N.P.) to remove artifacts (e.g., mentions of "4" in IFP #6 that referred to "Article 4" instead of a 4 percent probability of the event occurrence) and extract numerical estimates that were conveyed as linguistic expressions rather than actual numbers. For example, the exchange below:

- "I think it's 40 percent"

- "I agree"

was translated as two numerical estimates (40,40). To avoid experimenter's bias, we agreed in advance on the following translation rules:

- Expressions of low confidence such as "I think", "I believe", "maybe", "low/high" etc. were translated into ratings of 80% and 20%
- Expressions of high confidence such as "I'm sure", "definitely", "no way", "very low/very high" etc. were translated into ratings of 90% and 10%
- Expressions of total uncertainty like "no clue", "I have no idea", etc. were translated to 50%
- Expressions mentioning two numbers like "Between 10 and 20" were translated as the average between them
- Expressions of agreement were matched to the corresponding estimate (see example above)
- Whenever indecisive we abstained from entering an estimate

We computed the absolute distance between each numerical suggestion and the final consensus estimate entered by each group to each IFP. We called this measure "convergence" as it represent the convergence of opinions from initial private judgments to final group forecasts. This convergence measure has an upper bound of 100, representing the maximum disagreement possible (on a percentage scale) between a suggested estimate and the final consensus. A value of 0 represents the consensus estimate the group agrees upon. Supplementary Figure 6 shows the mean absolute convergence (i.e., absolute distance between each estimate and the final consensus forecast) for each experimental condition. It can be seen that numerical estimates in small diverse groups showed greater variability (on a average) from the consensus forecast, in accordance with our disagreement analysis. The graph is suggesting that in small diverse groups (compared to other experimental conditions), estimates mentioned during the online discussion were noisier and further away from what the group ended up agreeing on. To analyze why this was the case, we turned to the fine grained numerical estimates over the course of conversation time (i.e., number of messages sent over the total number of messages sent, for each conversation). Each message sent within the chat was taken as a unit measure of information exchanged. Verbal exchange happened for different groups at different times (some conversations spanning the entire 4.5 seconds, others concentrated in small time frames). We ranked each message on a "conversation time" percentage scale, where 0 represent the beginning of the chat (before any message is sent) and 1 represents the last message sent, for each group and question. This measure represents time in conversational units of information (i.e., messages exchanged). Conversation time was preferred over a simple time measure, as it made comparisons across groups and conditions more meaningful and better interpretable. We modeled convergence over conversation time by fitting a sigmoid curve (`SSlogis, nls2` in `R`) of the form:

$$y = \frac{asym}{1 + e^{(x_{mid} - x)/\sigma}} \tag{1}$$

where the parameter $asym$ controls the upper asymptote, X_{mid} controls the inflection point and σ is a scaling parameter. We then used the inflection point values X_{mid} as a proxy for consensus reaching speed. Lower value indicate that consensus was reached early on in the conversation, while greater values indicate slower consensus reaching. We then fitted different alternative linear mixed-effect models to inflection values and compared them using AIC. The winning model included fixed effects for each experimental condition and an interaction term between the two, plus a random effect for question (Formula: $X_{mid} = div * mod + (1|question)$). Results are reported in Supplementary Table 16 and visually represented as a partial residuals plot in Supplementary Figure 7. We found a significant effect of diversity ($\beta = -0.31, SE = 0.12, t = -2.55, p = 0.1$), indicating that large diverse groups showed faster consensus reaching times than large homogeneous groups, and a significant effect of modularity ($\beta = -0.46, SE = 0.10, t = -4.68, p$¡.001), indicating that small homogeneous groups showed quicker consensus reaching than large homogeneous groups. Importantly, a significant interaction was found between the two terms ($\beta = 0.69, SE = 0.17, t = 4.004, p < .001$), suggesting that small diverse groups tended to reach consensus more slowly than large diverse groups or small homogeneous ones. The results confirmed the hypothesis that diversity differentially affects communication in small and large groups.

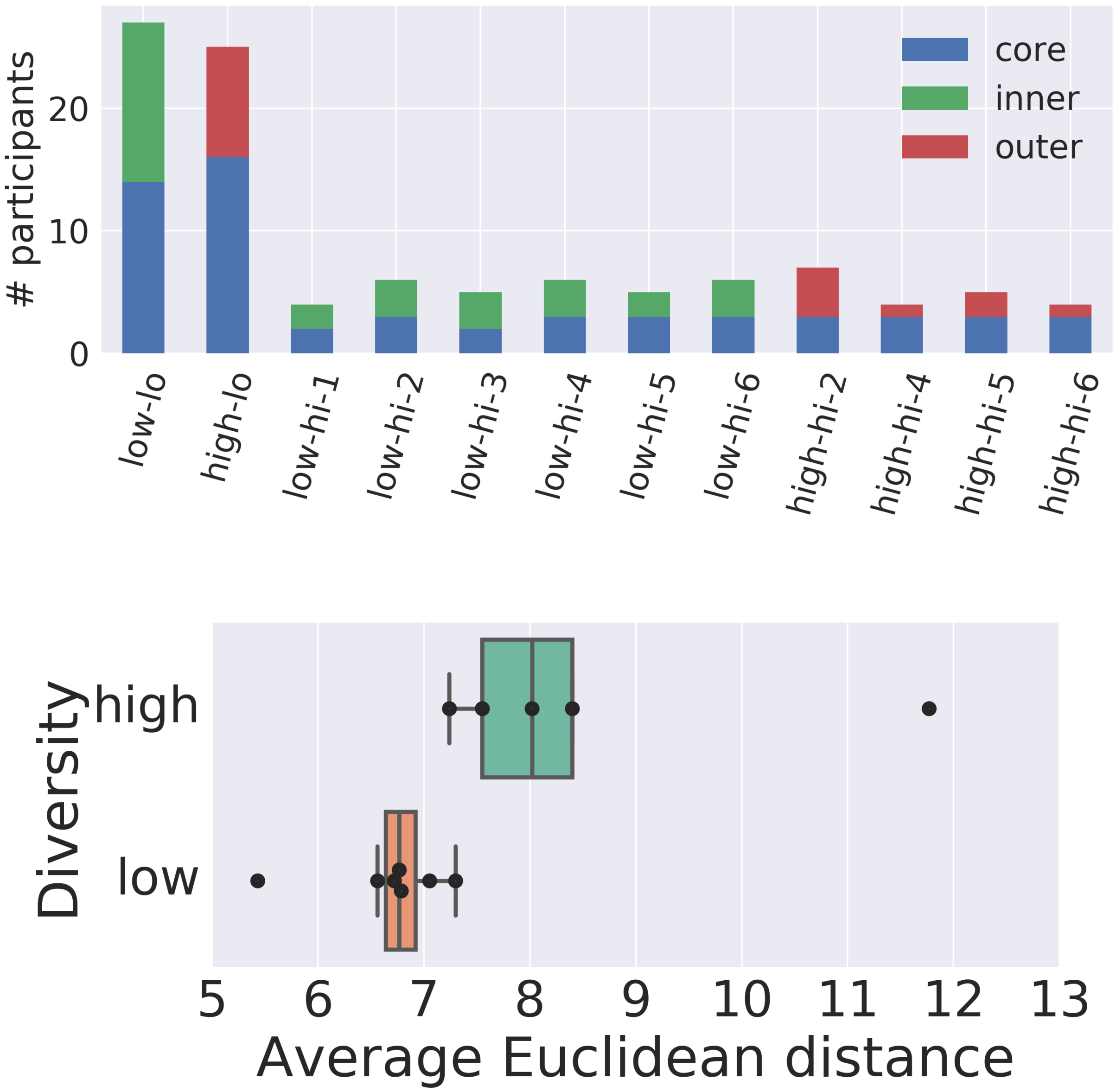

Supplementary Figure 1: Manipulation check. Upper panel: Partitions of test and treatment participants into different groups. Notice that two High-Hi groups were removed due to lack of participants in either the test or the treatment segment. Furthermore, test and treatment participants were fairly balanced within each group. Bottom panel: The experimental method was successful in manipulating the average diversity of the groups (n=12). Individuals in the High diversity condition showed a significantly larger average Euclidean distance with their team members, compared to individuals in the Low diversity condition. Box plots indicate median, inter-quartile range (IQR), 1.5 * IQR (whiskers) and data points outside the 1.5 * IQR range (outliers).

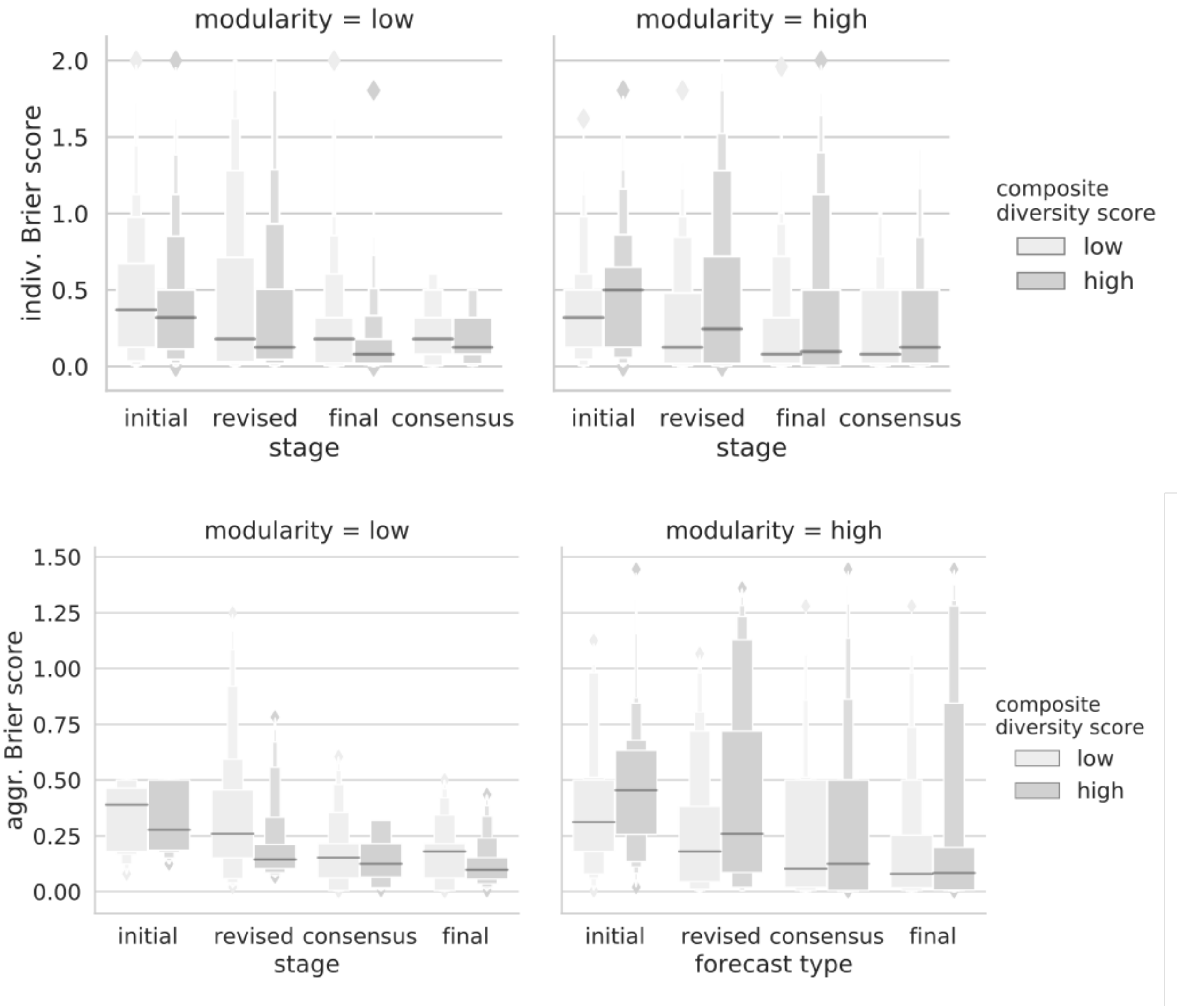

Supplementary Figure 2: Distributions of Brier error scores. Distributions of Brier error scores divided by condition and forecasting stage, for both individual forecasts (upper panel, n=104) and within-group aggregated forecasts (lower panel, n=12). Although diversity is beneficial in large groups (low modularity), it damages forecast accuracy in smaller groups (high modularity). Box areas correspond to distribution ideal tail areas of .50, .25, .125, .0625 [18].

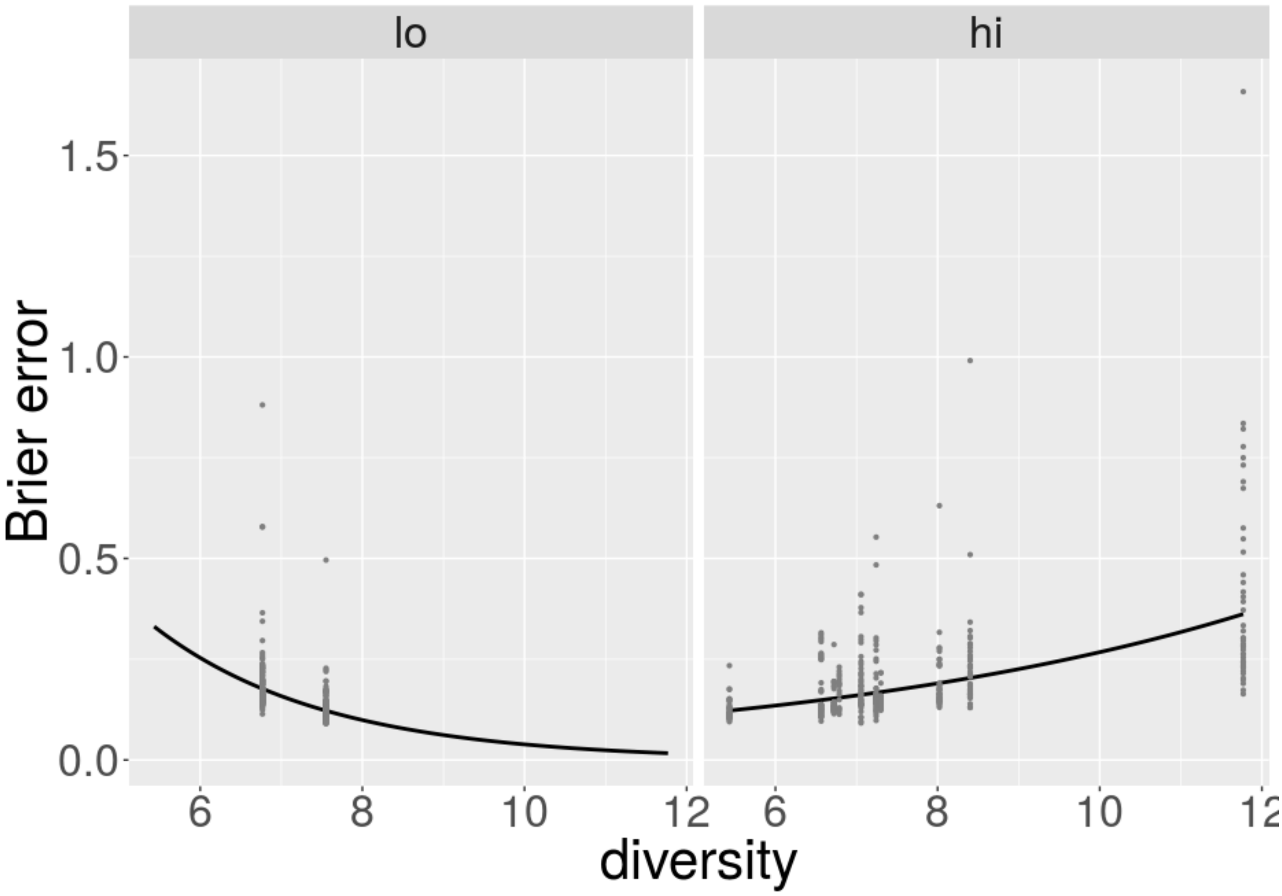

Supplementary Figure 3: Brier score as a function of continuous composite diversity score. Generalized linear mixed effect model using a continuous composite diversity score, namely group average Euclidean distance of its members, instead of diversity as binary factor.

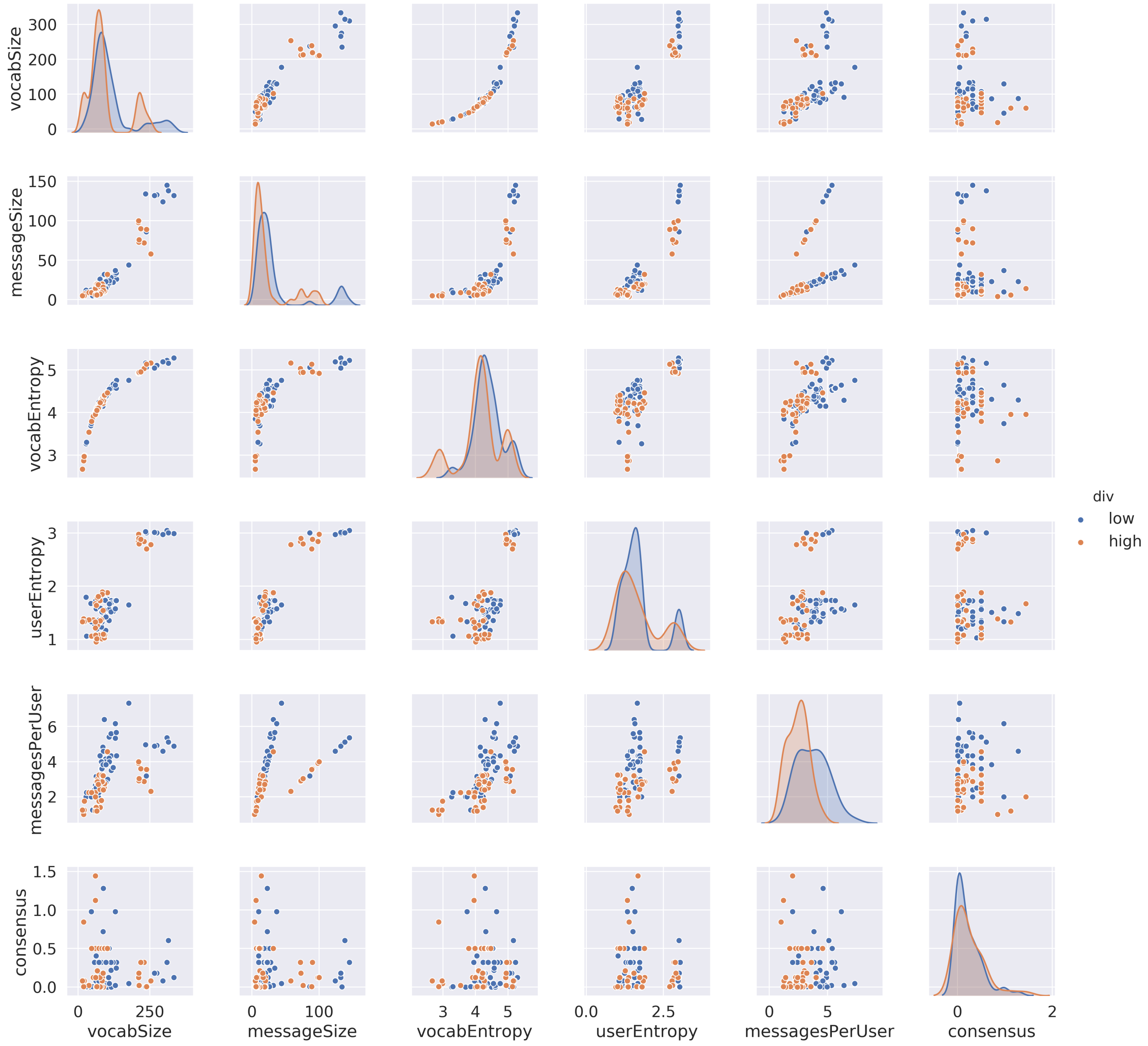

Supplementary Figure 4: Pairplot correlations of linguistic measures by diversity. Five different linguistic measures considered and divided by each experimental factor (color coded). Measures were computed for each group and individual forecasting problem (IFP) considered. Linguistic measures are divided according to our Composite Diversity manipulation.

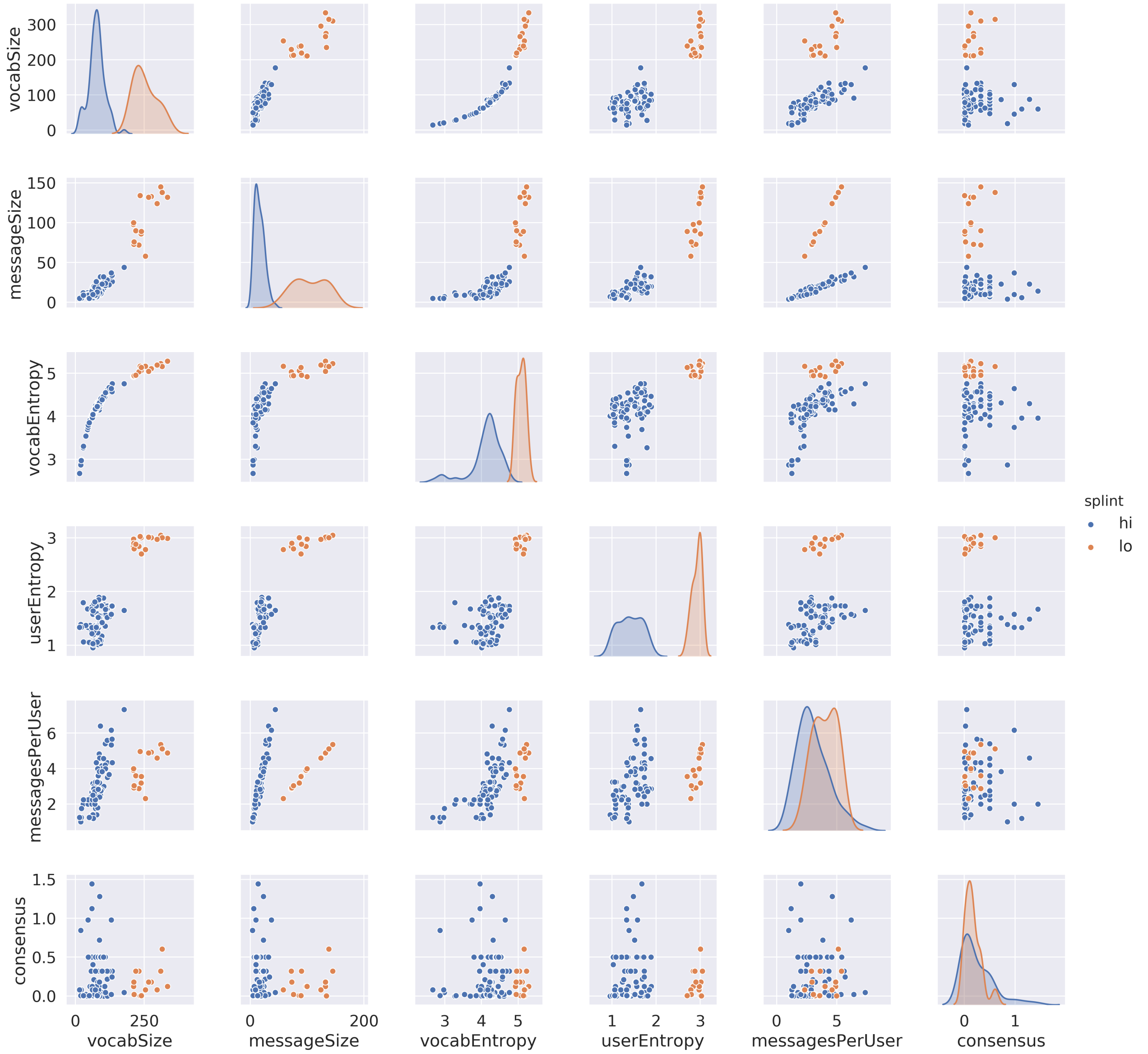

Supplementary Figure 5: Pairplot correlations of linguistic measures by modularity. Five different linguistic measures considered and divided by each experimental factor (color coded). Measures were computed for each group and individual forecasting problem (IFP) considered. Linguistic measures are divided according to our Modularity manipulation.

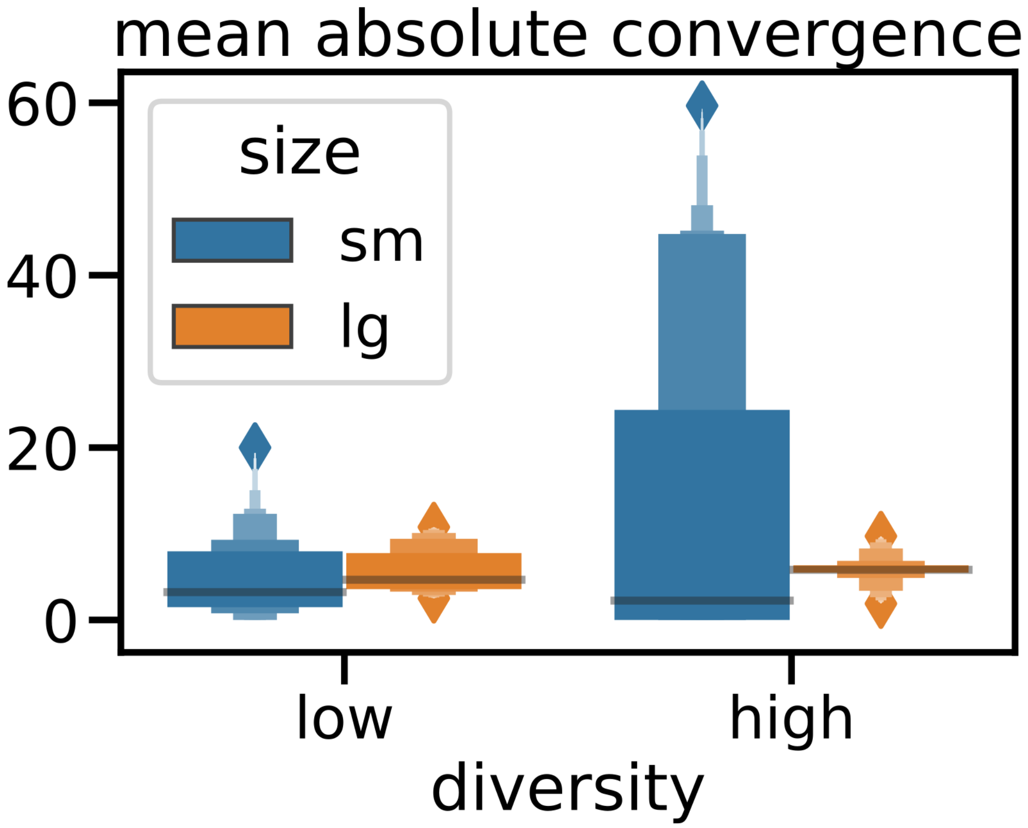

Supplementary Figure 6: Mean absolute distance between individual estimates and consensus. Mean absolute distance between group members' numerical estimates in the conversation and consensus forecast (n=8 for large groups; n=32 for diverse small groups; n=48 for homogeneous small groups. Box areas correspond to distribution ideal tail areas of .50, .25, .125, .0625 [18].

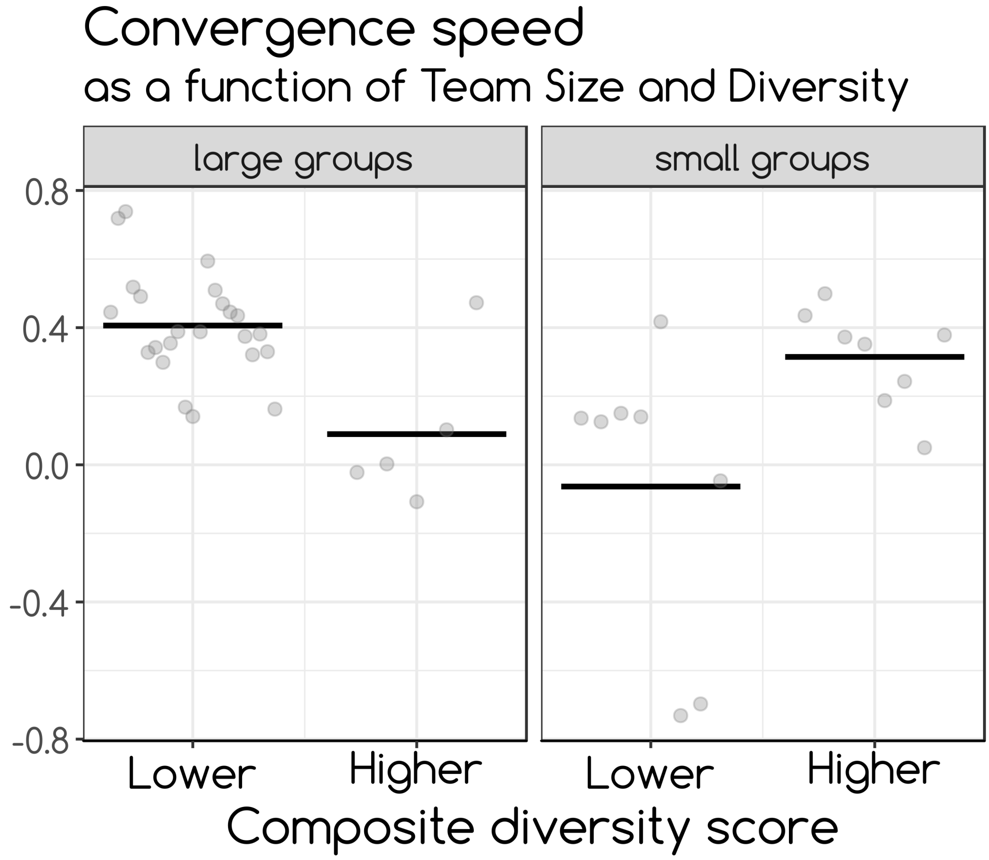

Supplementary Figure 7: Convergence speed to consensus. Partial residual plot of convergence speed as a function of diversity and modularity. For each conversation (each group and question), a sigmoid curve was fitted to the convergence data (i.e., distance of each numerical estimate from consensus) and the inflection point X_{mid} was taken as a measure of convergence speed. Lower values X_{mid} represent conversations when consensus was reached more quickly. A linear mixed-effects model with formula: $X_{mid} = div * mod + (1|questions)$ was fitted to the inflection points. Black lines represent fitted values. The model shows the interaction between the two experimental factors, suggesting that small diverse groups tend to reach consensus more slowly than small homogeneous groups. The same is not true for large groups, where diversity has the opposite effect. See Supplementary Table 16 for reference.

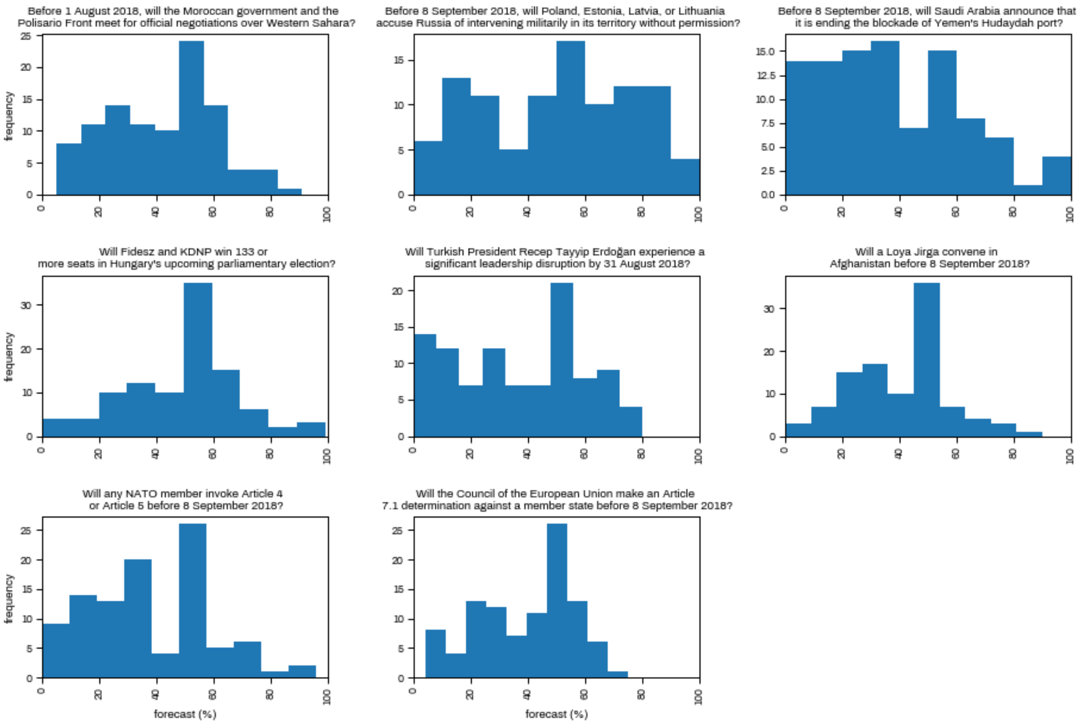

Supplementary Figure 8: Distribution of forecasts across forecasting problems. Although seven out of eight forecasting problems had a negative outcomes (did not happen), participants' forecasts were equally distributed across the probability spectrum (x-axis) on each question, indicating that they were insensitive to this bias. E.g., no question showed a highly skewed distribution on 0 (event surely won't happen) or 1 (event will surely happen), suggesting that participants were uncertain about the problem's outcome.

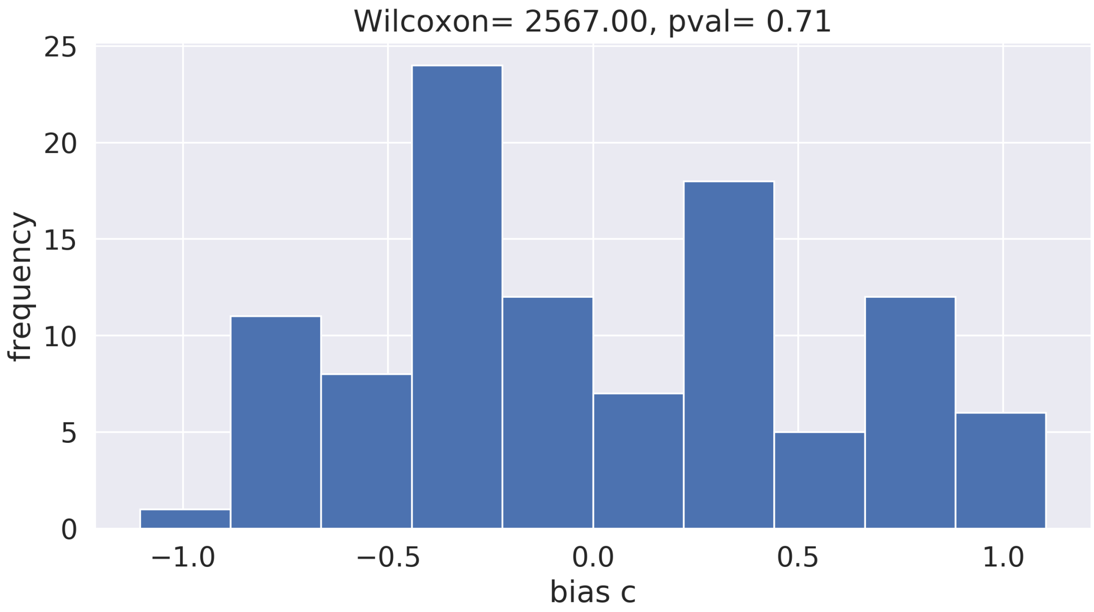

Supplementary Figure 9: Detection criterion distribution across participants. Signal detection theoretical analysis of participant's bias (criterion c) (n=104). Although seven out of eight forecasting problems had negative outcomes (did not happen), participants' forecasts did not show shift in bias. In other words they were equally distributed across the probability spectrum, indicating that they were not knowledgeable of this bias. P-value represents two-sided hypothesis test.

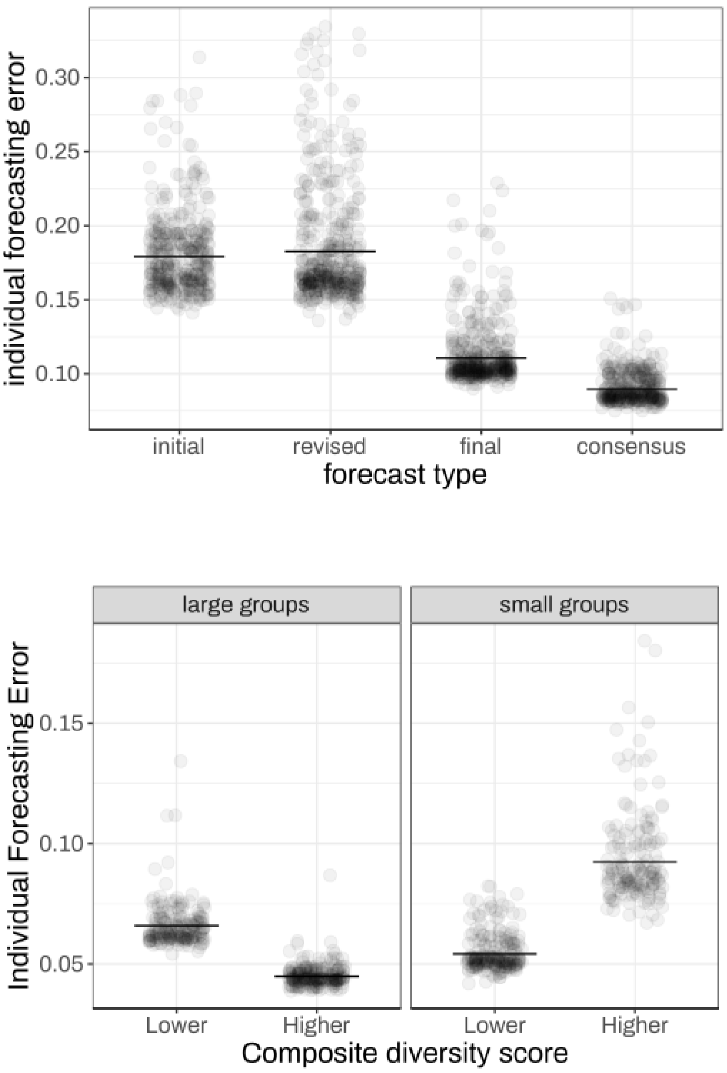

Supplementary Figure 10: Forecasting error as a function of forecast type and condition. Formulas: $brier \sim type + (1|Id : groupId) + (1|Q)$ and $brier \sim type + div * mod + (1|Q)$. Same as Figure 2 in the main text with a family=gaussian(link=logit). See Supplementary Table 17 for statistical results.

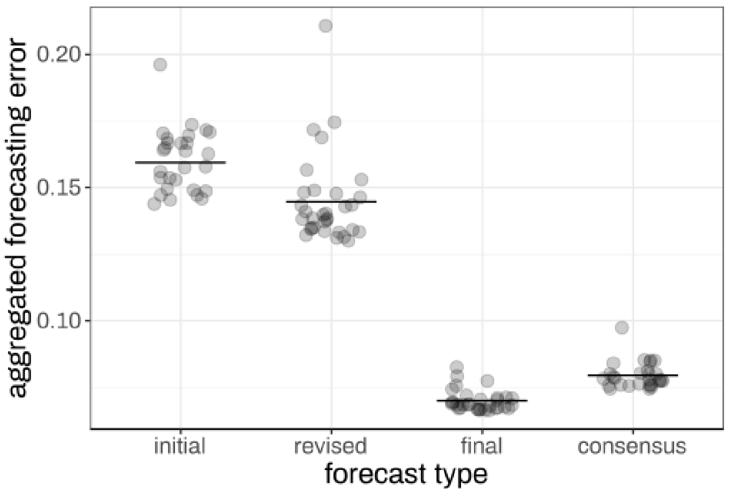

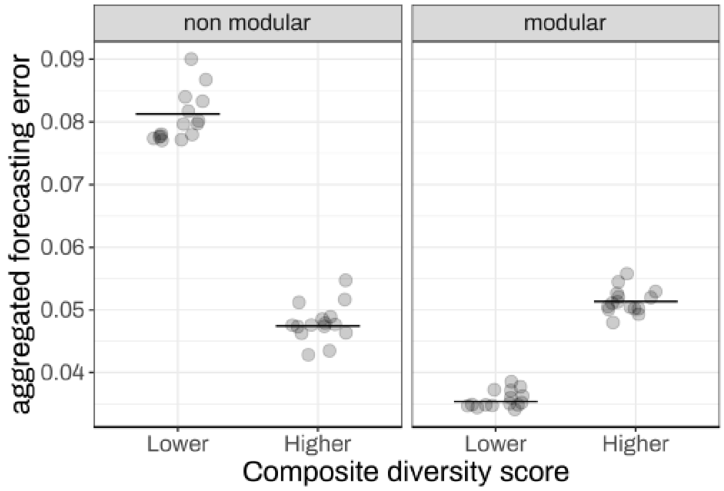

Supplementary Figure 11: Aggregated forecasting error as a function of forecast type and condition. Formulas: $brier \sim type + (1|Id : groupId) + (1|Q)$ and $brier \sim type + div * mod + (1|Id : groupId) + (1|Q)$. Same as Figure 3 in the main text with a family=gaussian(link=logit). See Supplementary Table 17 for statistical results.

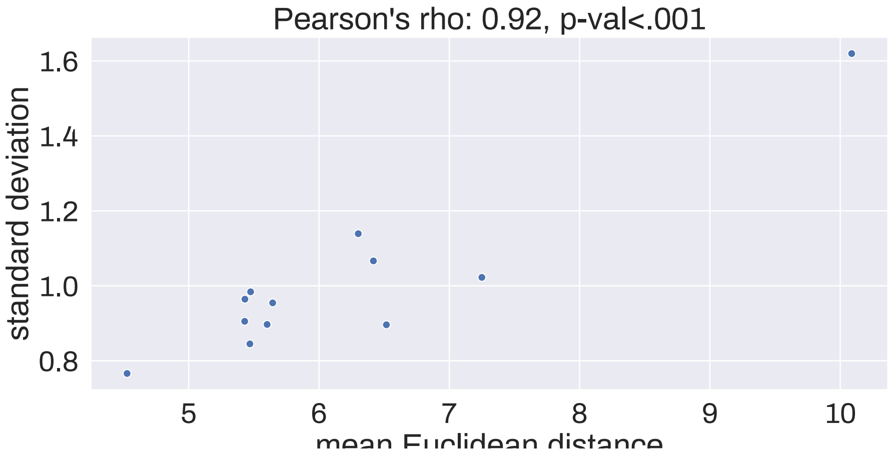

Supplementary Figure 12: Relation between Euclidean distance and standard deviation. Euclidean distance and standard deviation are two common measures of group diversity. The plot shows that these two measures are highly correlated in our experiment. This suggests that the results were unlikely to be dependent on the particular diversity measure chosen in the experiment (Euclidean distance).

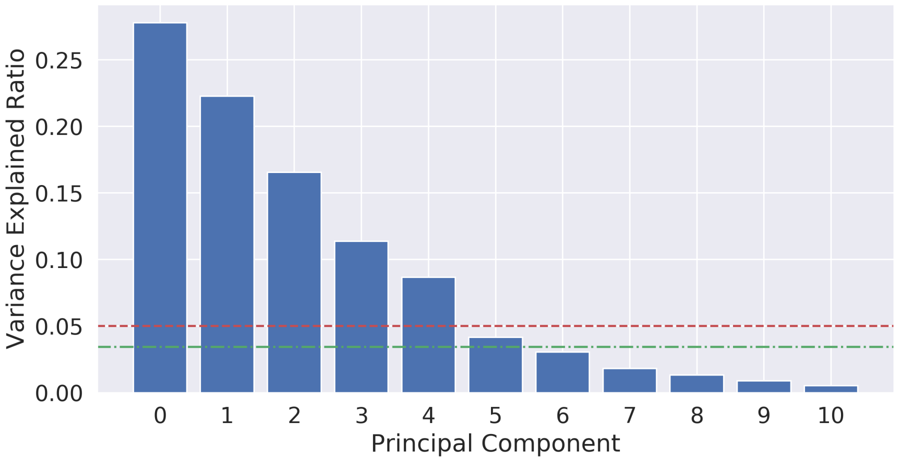

Supplementary Figure 13: Principal component analysis of questionnaire data. Five principal components seem to explain most of the variance found in the pre-screening data.

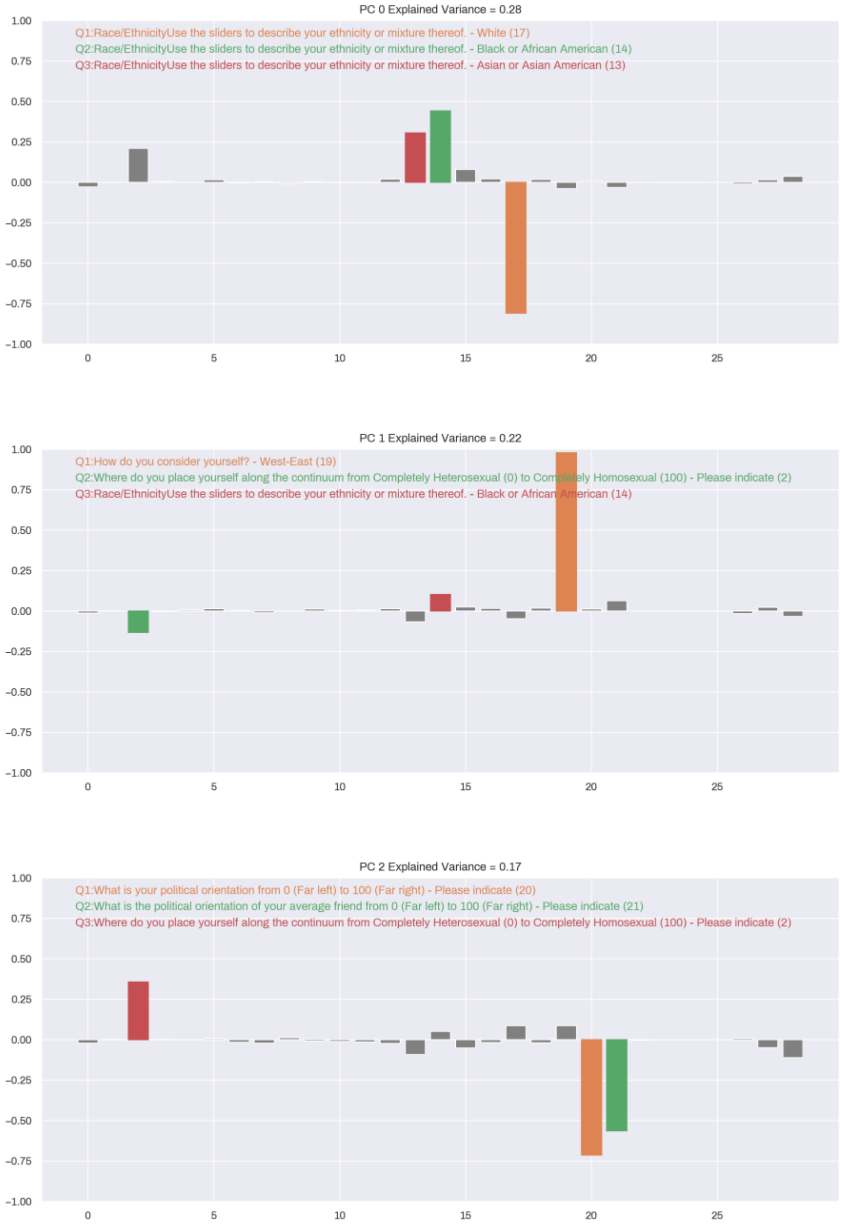

Supplementary Figure 14: Pre-test questions eigenvectors along the first three principal components. The three questions with the highest eigenvector values are colored. The first PC seems to capture variance related to race/ethnicity (white-non white dimension), the second one East-West culture block, the third one political orientation.

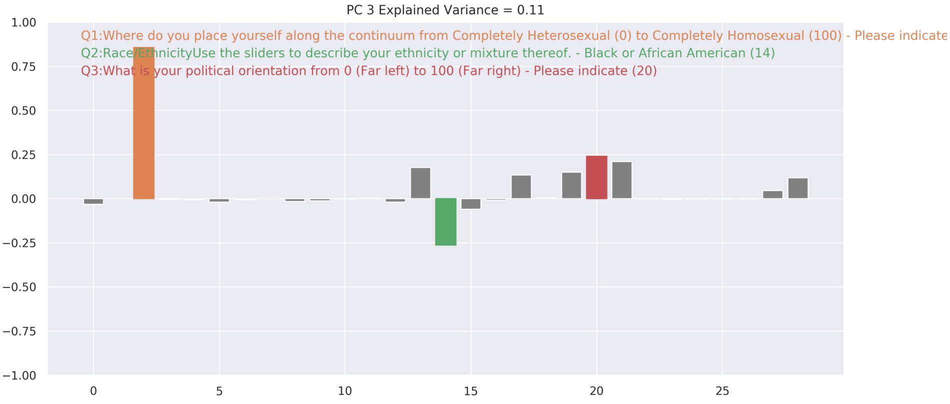

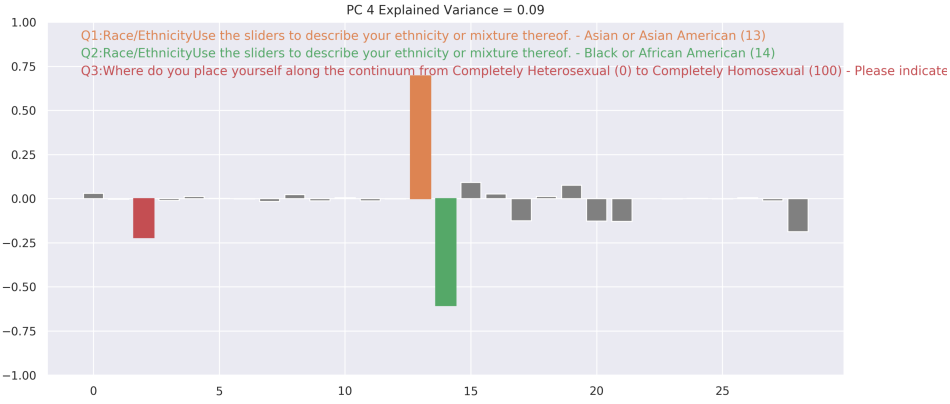

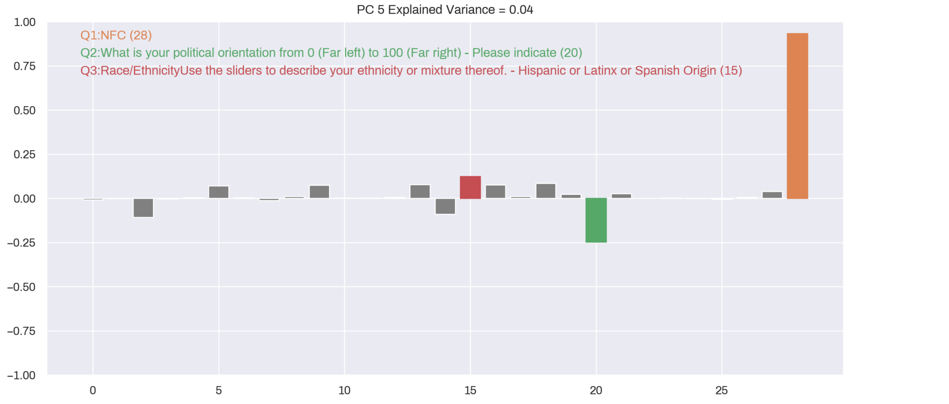

Supplementary Figure 15: Pre-test questions eigenvectors along the fourth to sixth principal components. The three questions with the highest eigenvector values are colored. The fourth PC seems to capture variance related to sexual orientation, the fifth PC variance related to race/ethnicity (Asian-black dimension) and the sixth need for cognition.

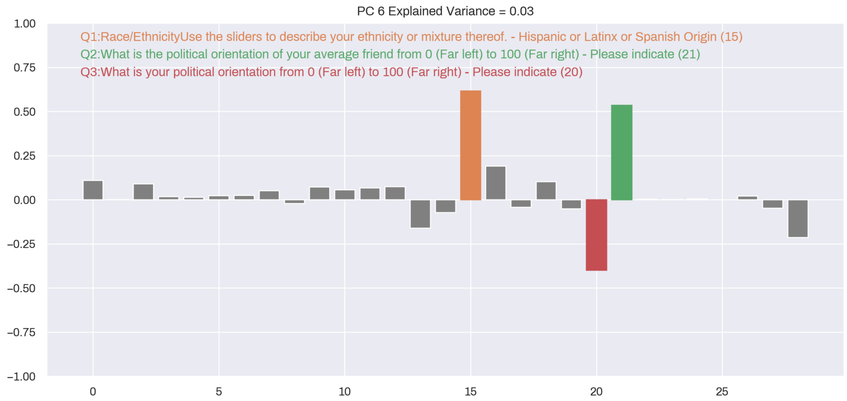

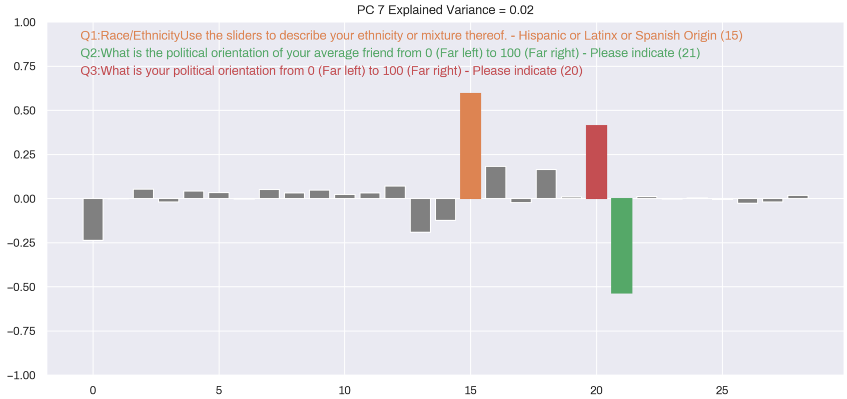

Supplementary Figure 16: Pre-test questions eigenvectors along the seventh and eighth principal components. The three questions with the highest eigenvector values are colored. The seventh and eighth PCs seem to capture variance related to race/ethnicity (Hispanic dimension) and political orientation (left-right wing).

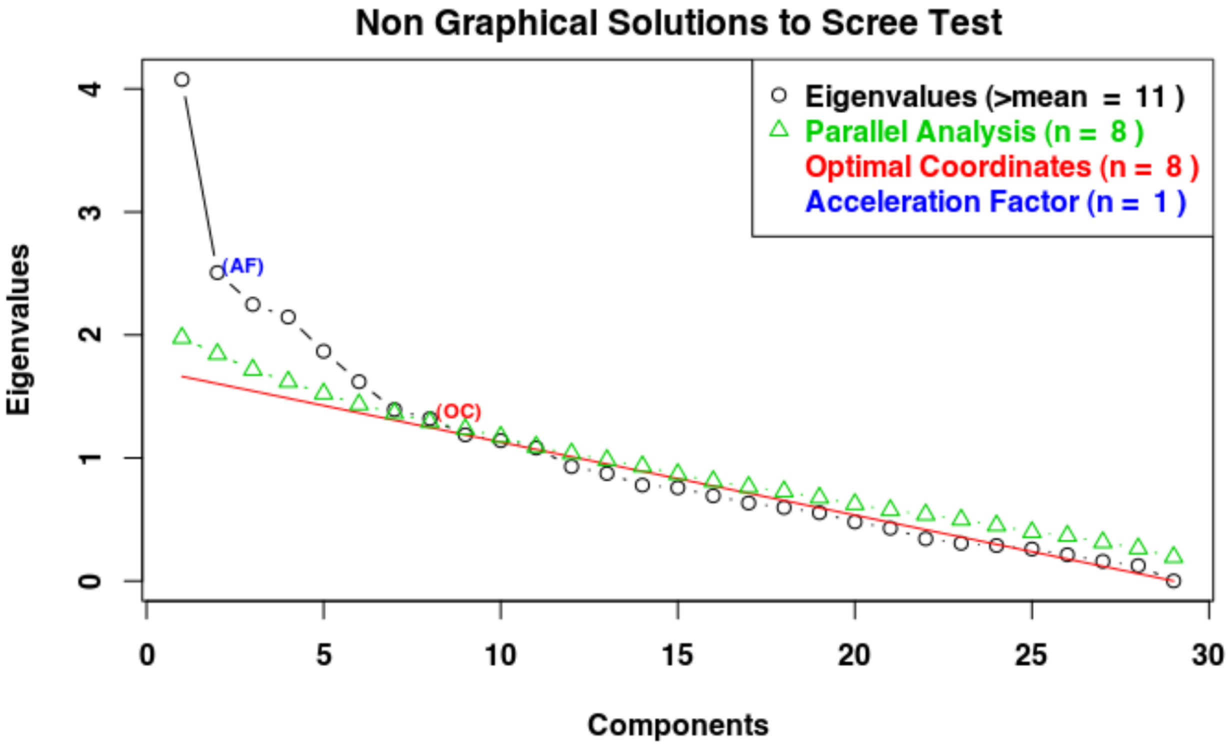

Supplementary Figure 17: Data-driven selection of number of principal components. Non graphical solutions to screen-plot. One PC is selected using the acceleration criterion (the elbow of the curve), while 8 are selected using parallel analysis.

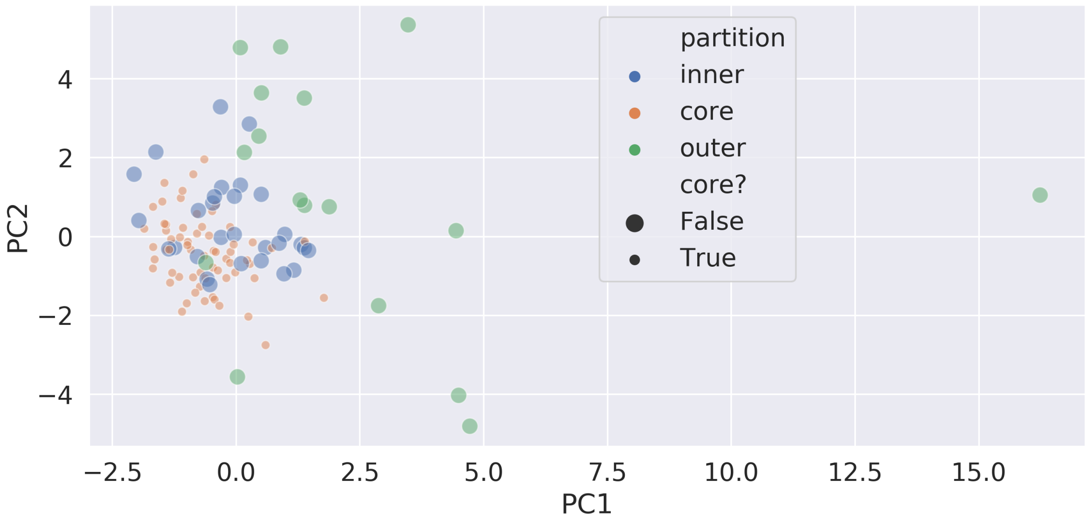

Supplementary Figure 18: Two dimensional projection from Theta space to PC space. Participants segmentation can already been observed onto the low-dimensional projection, with core participants being closer to inner participants than to outer participants. These two first principal components, explained about 40-50 percent of the variance. Randomizing participants in the core segment to interact with participants belonging to the one of the remaining segments is likely to have produced groups that differed based on their proportion of white and non-white individuals.

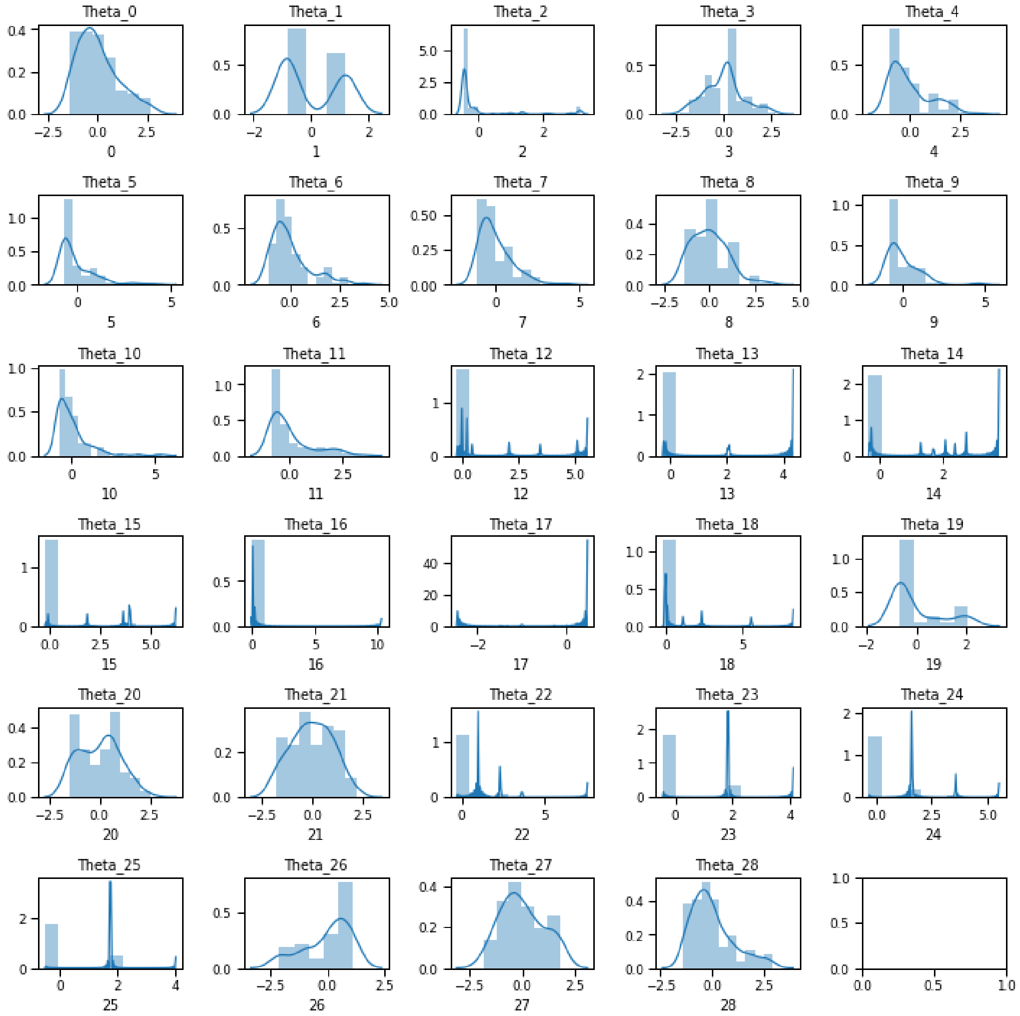

Supplementary Figure 19: Pretest question response distributions for each of the 29 questions asked. Most of the 29 questions asked showed good variability of response across participants.

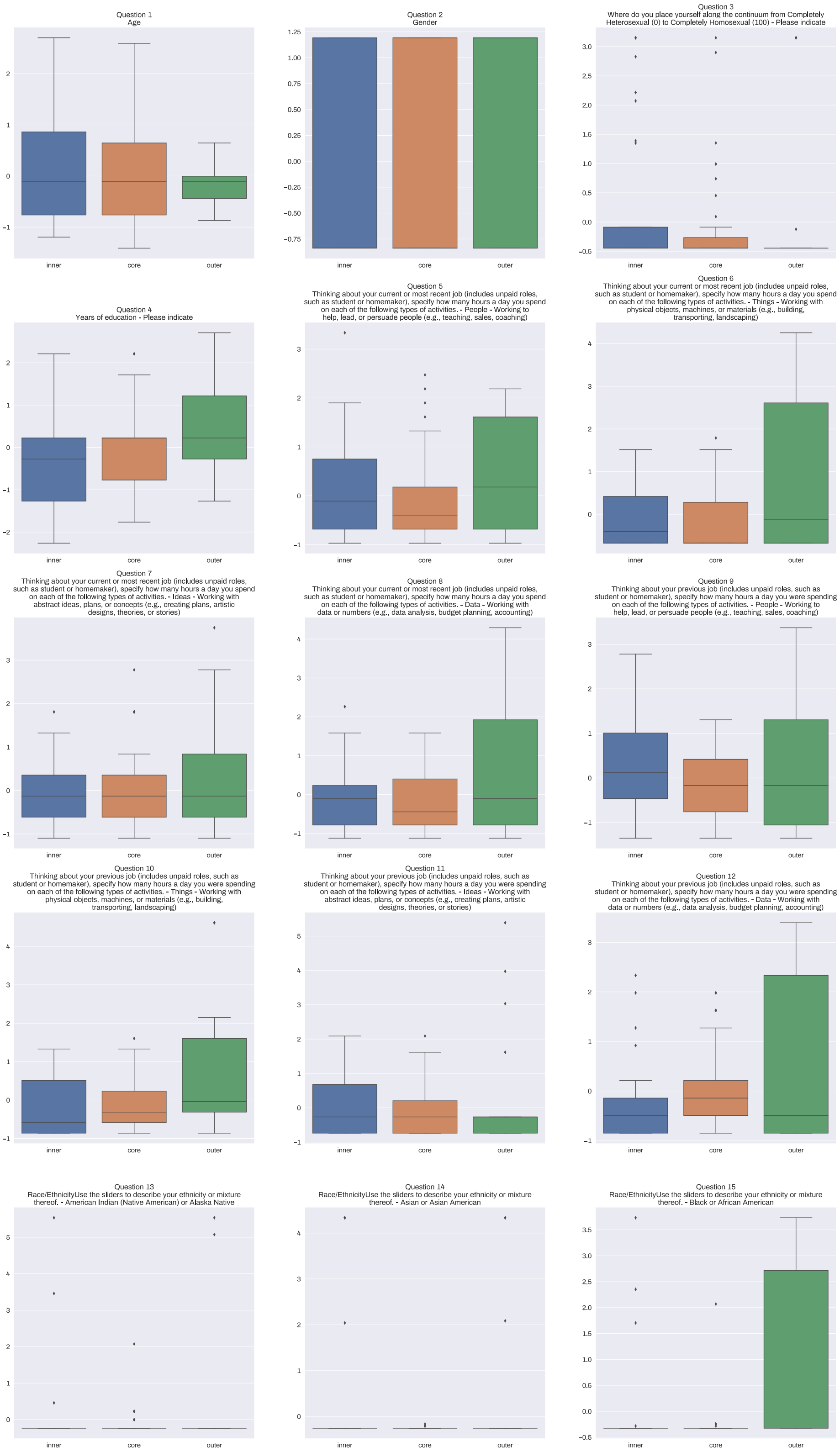

Supplementary Figure 20: Questionnaire responses by population segment. Pretest question response distribution broken down by population segment (Core, Inner, Outer). N=109. Box plots indicate median, inter-quartile range (IQR), 1.5 * IQR (whiskers) and data points outside the 1.5 * IQR range (outliers).

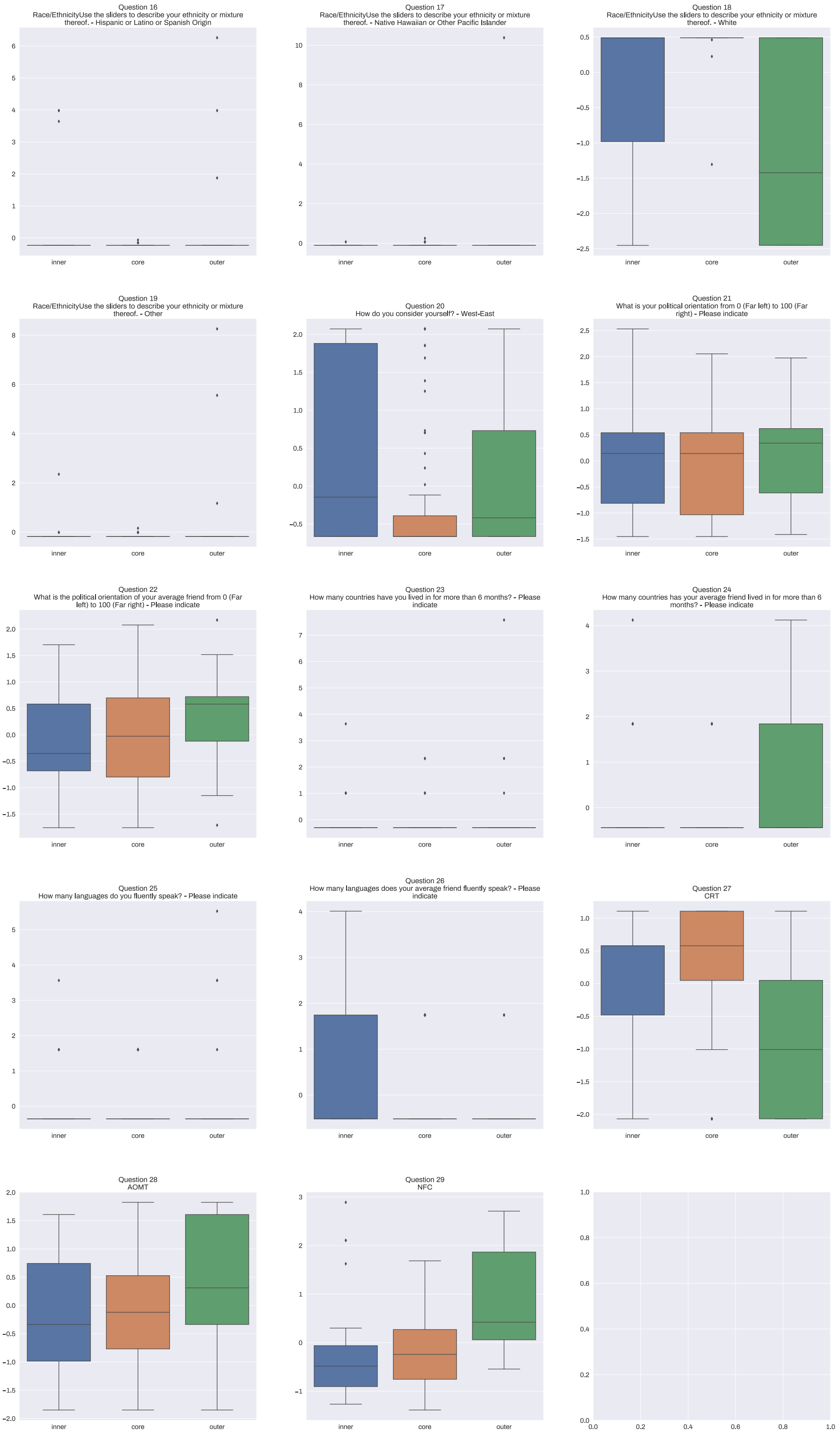

Supplementary Figure 21: Questionnaire responses by population segment. Pretest question response distribution broken down by population segment (Core, Inner, Outer). N=109. Box plots indicate median, inter-quartile range (IQR), 1.5 * IQR (whiskers) and data points outside the 1.5 * IQR range (outliers).

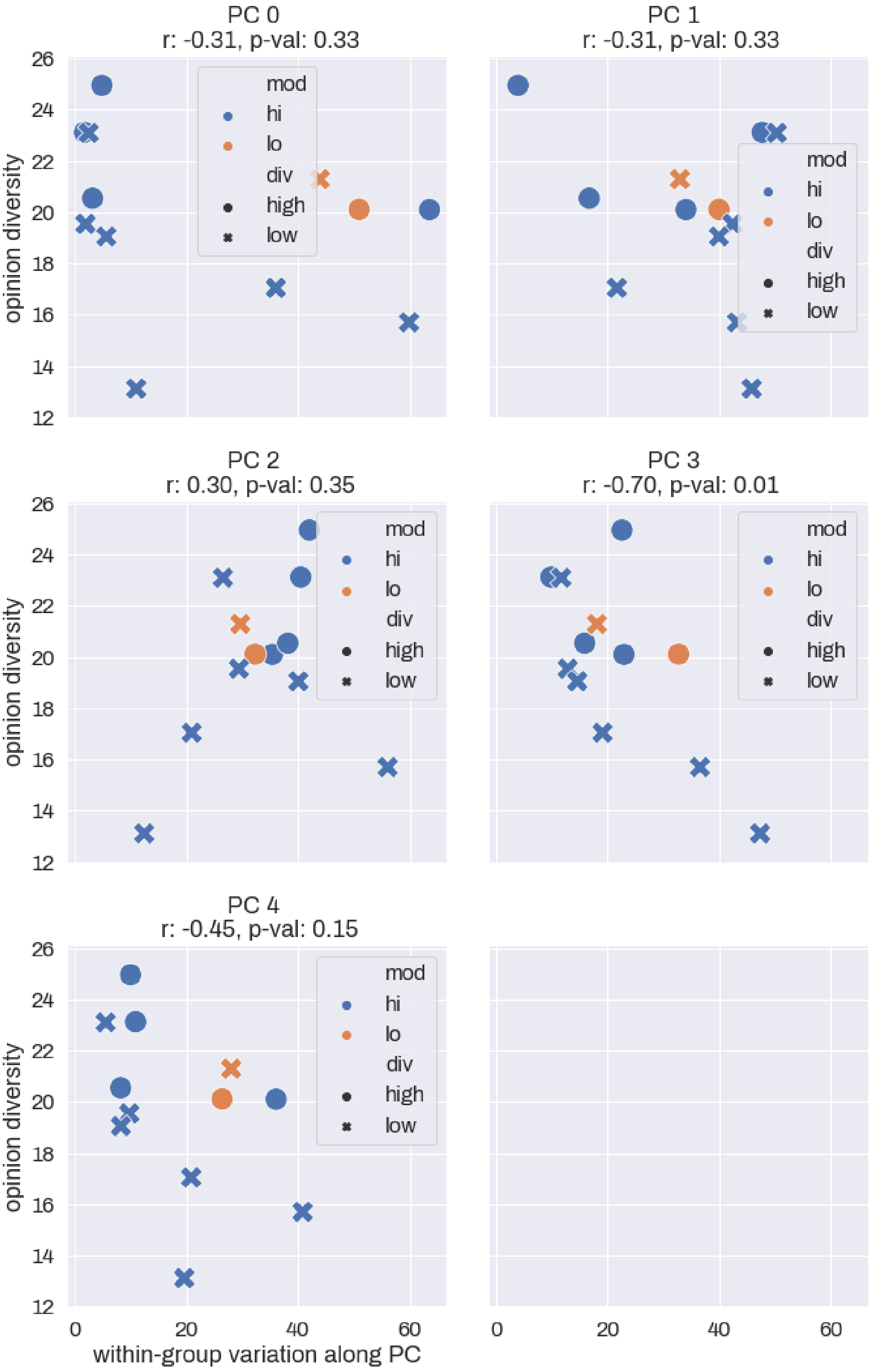

Supplementary Figure 22: Variability in forecasts and variability in pre-test questionnaire responses. Within-group opinion diversity (measured as median standard deviation of initial forecasts) as a function of within-group variability along each principal components, broken down by modularity (color) and diversity (shape) condition. The number of data points for each condition is reported in Supplementary Table 3. Principal components were computed on pre-test questionnaire responses (Θ) and roughly captured variability in participants' ethnicity (PC0 and PC4), East-West culture (PC1), political orientation (PC2), sexuality (PC3). Notice: (a) the wider variation in opinion diversity across small (blue) rather than large (orange) groups; (b) homogeneous small groups (blue crosses) tend to show lower opinion diversity (y-axis) than diverse large groups (blue circles). No hypothesis testing is used in this graph.

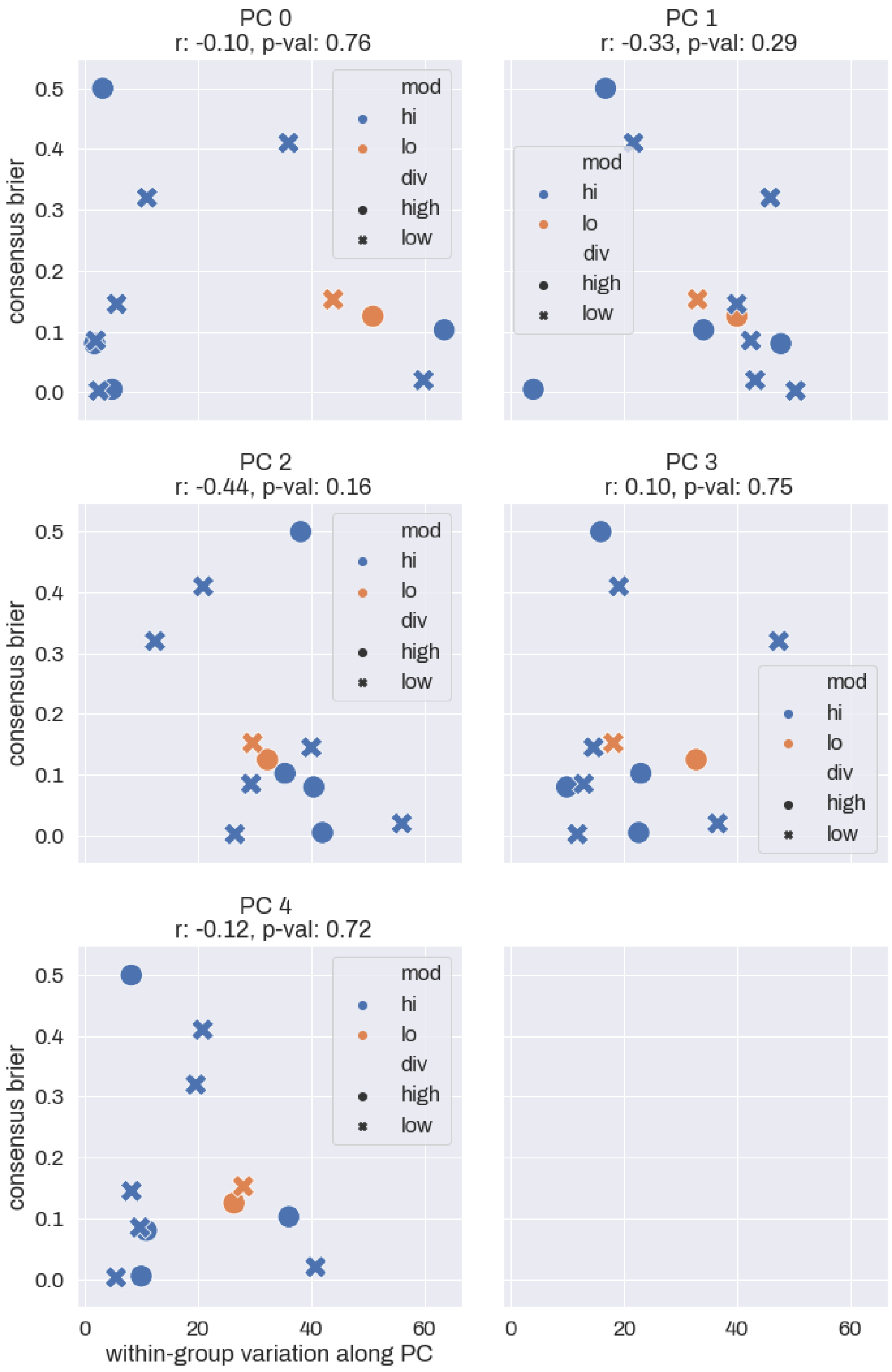

Supplementary Figure 23: Variability in forecasts and variability in pre-test questionnaire responses. Within-group median consensus forecasting error (brier score) as a function of the within-group variability along each principal components, broken down by modularity (color) and diversity (shape) condition. The number of data points for each condition is reported in Supplementary Table 3. Principal components were computed on pre-test questionnaire responses (Θ) and roughly captured variability in participants' ethnicity (PC0 and PC4), East-West culture (PC1), political orientation (PC2), sexuality (PC3). A wider spread of forecasting errors is observed in small groups (blue) than large groups (blue color), suggesting greater noise. While the large diverse group shows reduced error than large homogeneous group (orange cross vs. dot), the relation is less straightforward in small groups (blue crosses vs. dots). No hypothesis testing is used in this graph.

Individual Forecasting Problems (IFPs)	Truth revealed	Ground truth
**1.** Before 1 August 2018, will the Moroccan government and the Polisario Front meet for official negotiations over Western Sahara?	2018-08-03	0
**2.** Before 8 September 2018, will Poland, Estonia, Latvia, or Lithuania accuse Russia of intervening militarily in its territory without permission?	2018-09-10	0
**3.** Before 8 September 2018, will Saudi Arabia announce that it is ending the blockade of Yemen's Hudaydah port?	2018-09-10	0
**4.** Will Fidesz and KDNP win 133 or more seats in Hungary's upcoming parliamentary election?	2018-04-11	1
**5.** Will a Loya Jirga convene in Afghanistan before 8 September 2018?	2018-09-10	0
**6.** Will any NATO member invoke Article 4 or Article 5 before 8 September 2018?	2018-09-10	0
**7.** Will the Council of the European Union make an Article 7.1 determination against a member state before 8 September 2018?	2018-09-10	0
**8.** Will Turkish President Recep Tayyip Erdoğan experience a significant leadership disruption by 31 August 2018?	2018-09-04	0

Supplementary Table 1: Individual forecasting problems. All IFPs were formulated within the IARPA HFC tournament, and thus represent independent decision-problems. Ground truths were revealed by the IARPA HFC tournament (hence also independently from experimenters' biases) and on the dates specified above (YYYY-MM-DD format). Ground truths are represented on the right column: 0 = the event did not occur; 1 = the event did occur. Question order was randomized for each group. Distribution of forecasts across questions and signal detection theoretical analysis of response bias is provided to show that the results cannot be explained by a general tendency for low probabilities (Supplementary Figures 8-9).

	Diversity	
**Modularity**	Low	High
Lo	N=46, M=1	N=51, M=1
Hi	N=46*, M=6	N=50**, M=6

Supplementary Table 2: Experiment 1 - Nominal participant partitions. DBSCAN was used to create one single large Core cluster (test participants) ad two smaller clusters (Inner and Outer periphery). Core participants were then randomly matched with Inner or Outer participants and solved the task in large (Lo modularity) or small (Hi modularity) groups. The table above shows the partitioning of 193 participants who responded to the original survey into the four cell of the factorial design. Participants in the Hi condition were divided in $M = 6$ groups containing *(8,8,8,8,8,6) and **(8,8,9,8,9,8) members respectively.

	Diversity	
**Modularity**	Low	High
Lo	N=27, M=1	N=25, M=1
Hi	N=32*, M=6	N=20**, M=4

Supplementary Table 3: Actual participant partitions. Many initial respondents did not return on the day of the main experiment ($\sim 41.9\%$ attrition rate). Furthermore, two High-Hi groups were removed because the did not pass the exclusion criteria (i.e. at least one test and one treatment participant in each group). The above table shows the final numbers. Participants in the Hi condition were divided in $M = 6$ groups containing *(4,6,5,6,5,6) and **(x,7,x,4,5,4) members, respectively. Xs refer to the groups that were removed.

**Effects**	Estimate	SE	z-value	p-value
Intercept	-2.14224	0.24230	-8.841	$<$ **2e-16**
Final	0.23849	0.09979	2.390	**0.0169**
Initial	0.62237	0.09040	6.884	**5.81e-12**
Revised	0.69532	0.08947	7.772	**7.73e-15**

Supplementary Table 4: Individual-level analysis: effect of forecast type. Formula: $brier \sim type + (1|X_{id} : group) + (1|Q)$ Same model as reported in Table 1A of the main text, with the addition of the GLMM formula. All tests refer to two-sided hypotheses and were calculated with the lmerTest package in R [19]. P-values are not adjusted for multiple comparisons.

**Effects**	Estimate	SE	z-value	p-value
Intercept	-1.75634	0.29911	-5.872	**4.31e-09**
Final	0.20997	0.07814	2.687	**0.00720**
divHigh	-0.37285	0.20278	-1.839	*0.06595*
modHi	-0.20011	0.20094	-0.996	0.31932
divHigh:modHi	0.82955	0.29025	2.858	**0.00426**

Supplementary Table 5: Individual-level analysis: effect of condition (stage 3 forecasts only). Formula: $brier \sim type + div * mod + (1|X_{id} : group) + (1|Q)$ Same model as reported in Table 1B of the main text, with the addition of the GLMM formula. All tests refer to two-sided hypotheses and were calculated with the lmerTest package in R [19]. P-values are not adjusted for multiple comparisons.

**Effects**	Estimate	SE	t-value	p-value
Intercept	1.23681	1.75014	0.707	0.47976
typefinal	0.20960	0.07815	2.682	**0.00732**
MED	-0.46981	0.24018	-1.956	*0.05045*
modHi	-4.47469	1.77770	-2.517	**0.01183**
MED:modHi	0.64080	0.24688	2.596	**0.00944**
(Intercept)	1.6968	2.0059	0.846	0.39760
typefinal	0.2088	0.0782	2.670	**0.00757**
MED_N	-4.4747	2.3131	-1.935	*0.05305*
modHi	-4.6159	2.0119	-2.294	**0.02177**
MED_N:modHi	5.5756	2.3457	2.377	**0.01746**

Supplementary Table 6: Effect of forecast type, euclidean distance and group size on forecasting error (Brier score). Top: To test that our conclusions held also using a non binary measure of diversity we replicated the analysis reported in the main text using a continuous measure of diversity, namely group average Euclidean distance among its members MED. Both diversity and modularity had positive effects on performance, but they affected each other negatively as indicated by the significant interaction found between the two terms (Supplementary Figure 3). Bottom: We replicate the analysis also applying group size correction (MED_N), suggested in [20], Equation 13. All tests refer to two-sided hypotheses and were calculated with the lmerTest package in R [19]. P-values are not adjusted for multiple comparisons.

**Effects**	Estimate	SE	z-value	p-value
Intercept	-1.92890	0.26862	-7.181	**6.94e-13**
divHigh	-0.16616	0.25766	-0.645	0.519
modHi	-0.01084	0.26126	-0.041	0.967
divHigh:modHi	0.51866	0.37165	1.396	0.163

Supplementary Table 7: Individual-level analysis: effect of condition on consensus forecasts. Formula: $brier \sim div * mod + (1|X_{id} : group) + (1|Q)$

**Effects**	Estimate	SE	z-value	p-value
Intercept	-1.7361	0.3386	-5.127	**2.94e-07**
divHigh	-0.4312	0.2441	-1.767	*0.07730*
modHi	-0.3015	0.2372	-1.271	0.20361
divHigh:modHi	0.9420	0.3426	2.750	**0.00597**

Supplementary Table 8: Individual-level analysis: effect of condition on final forecasts. Formula: $brier \sim div * mod + (1|X_{id} : group) + (1|Q)$ All tests refer to two-sided hypotheses and were calculated with the lmerTest package in R [19]. P-values are not adjusted for multiple comparisons.

**Effects**	Estimate	SE	z-value	p-value
Intercept	-1.8387	0.2508	-7.331	**2.29e-13**
Final	-0.1281	0.2964	-0.432	0.66557
Initial	0.6815	0.2293	2.972	**0.00296**
Revised	0.5999	0.2301	2.607	**0.00913**

Supplementary Table 9: Group-level analysis: effect of forecast type. Formula: $brier \sim type + (1|Q)$ Same model as reported in Table 1C of the main text, with the addition of the GLMM formula. All tests refer to two-sided hypotheses and were calculated with the lmerTest package in R [19]. P-values are not adjusted for multiple comparisons.

**Effects**	Estimate	SE	z-value	p-value
Intercept	-1.76627	0.33428	-5.284	**1.27e-07**
Final	-0.06877	0.15360	-0.448	0.65434
divHigh	-0.56382	0.23514	-2.398	**0.01649**
modHi	-0.82268	0.26515	-3.103	**0.00192**
divHigh:modHi	0.93267	0.38254	2.438	**0.01477**

Supplementary Table 10: Group-level analysis: effect of condition. Formula: $brier \sim type + div * mod + (1|Q)$. Same model as reported in Table 1D of the main text, with the addition of the GLMM formula. All tests refer to two-sided hypotheses and were calculated with the lmerTest package in R [19]. P-values are not adjusted for multiple comparisons.

**Effects**	Estimate	SE	z-value	p-value
Intercept	1.7772	0.4912	3.618	**0.000296**
Final	0.2639	0.1824	1.446	0.148112
Initial	-1.0700	0.1695	-6.313	**2.73e-10**
Revised	-0.6011	0.1701	-3.534	**0.000409**

Supplementary Table 11: Individual-level analysis: effect of forecast type on binarized accuracy. Formula: $acc \sim type + (1|X_{id} : group) + (1|Q)$. At the individual level, a difference between social and non-social forecast types was found also in binarized accuracy. Contrary to Brier score analysis however, no significant difference between Final and Consensus was found. All tests refer to two-sided hypotheses and were calculated with the lmerTest package in R [19]. P-values are not adjusted for multiple comparisons.

**Effects**	Estimate	SE	z-value	p-value
Intercept	2.9238	1.2184	2.400	**0.01641**
Final	0.2680	0.2001	1.339	0.18063
DivHigh	0.9701	0.5107	1.900	*0.05749*
ModHi	-0.2768	0.4817	-0.575	0.56550
DivHigh:ModHi	-1.8888	0.7100	-2.660	**0.00781**

Supplementary Table 12: Individual-level analysis: effect of experimental manipulation on binarized accuracy. Formula: $acc \sim type + div * mod + (1|X_{id} : group) + (1|Q)$, Control: glmerControl(optimizer = 'bobyqa', optCtrl = list(maxfun = 1e+05)). Also with binarized accuracy, we find at the individual level a marginally significant effect of Diversity and a significant interaction between the two experimental factors. All tests refer to two-sided hypotheses and were calculated with the lmerTest package in R [19]. P-values are not adjusted for multiple comparisons.

**Effects**	Estimate	SE	z-value	p-value
Intercept	3.3431	1.0485	3.188	**0.00143**
Final	0.1389	1.4382	0.097	0.92306
Initial	-1.7857	1.1349	-1.573	0.11563
Revised	-1.3571	1.1516	-1.178	0.23864

Supplementary Table 13: Group-level analysis: effect of forecast type on binarized accuracy. Formula: $acc \sim type + (1|Q)$. Forecasts were aggregated within each cell for different forecast types. No significant difference was found between different forecast types. All tests refer to two-sided hypotheses and were calculated with the lmerTest package in R [19]. P-values are not adjusted for multiple comparisons.

**Effects**	Estimate	SE	z-value	p-value
Intercept	2.6654	1.1149	2.391	**0.0168**
Final	0.1412	1.4474	0.098	0.9223
Initial	-1.8608	1.1531	-1.614	0.1066
Revised	-1.4044	1.1662	-1.204	0.2285
DivHigh	0.6471	0.8183	0.791	0.4291
ModHi	1.1277	0.9192	1.227	0.2199
DivHigh:ModHi	0.1003	1.5110	0.066	0.9471

Supplementary Table 14: Group-level analysis: effect of experimental manipulation. Formula: $acc \sim type + div * mod + (1|Q)$. Although in the main text (Brier scores analysis) we limit our analysis to social forecasts only, doing this at the aggregated level on binarized accuracy leads to numerically singular Hessian, thus making parameter estimation impossible. We thus include all data and report the interaction model for comparison with the main text. All tests refer to two-sided hypotheses and were calculated with the lmerTest package in R [19]. P-values are not adjusted for multiple comparisons.

**Effects**	Estimate	SE	t-value	df	p-value
(Intercept)	20.4502	1.9567	119.3479	10.451	< **2e-16**
typeFin	-4.4099	1.1842	275.0000	-3.724	**0.000238**
typeRev	5.0630	1.1842	275.0000	4.275	**2.63e-05**
divHigh	-0.4829	2.3684	275.0000	-0.204	0.838572
modHi	-3.5188	1.8089	275.0000	-1.945	*0.052757*
divHigh:modHi	7.1175	2.6034	275.0000	2.734	**0.006665**
(Intercept)	25.5131	1.9567	119.3479	13.039	< **2e-16**
typeFin	-9.4729	1.1842	275.0000	-7.999	**3.51e-14**
typeInit	-5.0630	1.1842	275.0000	-4.275	**2.63e-05**
divHigh	-0.4829	2.3684	275.0000	-0.204	0.83857
modHi	-3.5188	1.8089	275.0000	-1.945	*0.05276*
divHigh:modHi	7.1175	2.6034	275.0000	2.734	**0.00666**

Supplementary Table 15: Disagreement analysis. GLM with formula = $disagreement \sim type + div * mod + (1|question))$**.** Model associated with analysis reported in the main text. Upper table: type reference is Initial. Lower table: type reference is Revised. All tests refer to two-sided hypotheses and were calculated with the lmerTest package in R [19]. P-values are not adjusted for multiple comparisons.

**Effects**	Estimate	SE	df	t-value	p-value
Intercept	0.40614	0.07067	12.55048	5.747	**7.73e-05**
divHi	-0.31666	0.12374	35.55257	-2.559	**0.014901**
modHi	-0.46950	0.10015	33.60373	-4.688	**4.45e-05**
divHi:modHi	0.69465	0.17349	34.49956	4.004	**0.000314**

Supplementary Table 16: Effect of condition on consensus reaching speed. The consensus reaching process was modeled as a sigmoid curve fitting the convergence measure, namely the distance from consensus at each point in conversation time. A linear mixed-effects model with formula: $X_{mid} = diversity * modularity + (1|question)$ was fitted to the curve's inflection point. See Supplementary Figure 7 for visualization. X_{mid} thus can be used to represent the moment in each conversation (for each group and question) when consensus was reached. Conversations where consensus is difficult to reach (i.e., disagreement is difficult to resolve), will tend to have larger values of X_{mid}. All tests refer to two-sided hypotheses and were calculated with the lmerTest package in R [19]. P-values are not adjusted for multiple comparisons.

(A) Individual forecasting error as a function of forecast type

Effect	Estimate	SE	t	p
(Intercept)	-1.52229	0.10540	-14.443	**< 2e-16**
Revised	0.02296	0.07969	0.288	0.773
Final	-0.56156	0.09801	-5.729	**1.01e-08**
Consensus	-0.79539	0.11740	-6.775	**1.24e-11**

(B) Individual forecasting error as a function of condition

Effect	Estimate	SE	t	p
(Intercept)	-2.8949	0.4555	-6.356	**2.07e-10**
Final	0.2441	0.0954	2.559	**0.01049**
Diverse	-0.4066	0.2532	-1.606	0.10833
Small	-0.2087	0.2545	-0.820	0.41221
Diverse:Small	0.9820	0.3679	2.670	**0.00759**

(C) Aggregated forecasting error as a function of forecast type

Effect	Estimate	SE	t	p
(Intercept)	-2.4479	0.2773	-8.828	**< 2e-16**
Initial	0.7852	0.2545	3.085	**0.00204**
Revised	0.6710	0.2552	2.629	**0.00857**
Final	-0.1389	0.3215	-0.432	0.66576

(D) Aggregated forecasting error as a function of condition

Effect	Estimate	SE	t	p
(Intercept)	-2.35172	0.37499	-6.271	**3.58e-10**
Final	-0.07704	0.17630	-0.437	0.66210
Diverse	-0.57480	0.26204	-2.194	**0.02827**
Modular	-0.88081	0.29201	-3.016	**0.00256**
Diverse:Modular	0.96315	0.42055	2.290	**0.02201**

Supplementary Table 17: Re-analysis of Table 1 (main text) with a Family=Gaussian(link = Logit). Generalized mixed-effects models on individual and aggregated errors. Table of analysis on forecasting errors (in halved Brier scores) for individual (A-B) and aggregated measures (C-D) and as a function of forecast type (A-C) and condition (B-D). Log-normal distributions used in the main text are common to model errors, but they have support $x \in [0, \infty]$. Instead, Brier scores are bounded in the range [0,2]. Compared to Table 1 in the main text, we here model halved Brier scores $\frac{1}{2}b$ using a *logit* link function, which has support $x \in [0, 1]$. This ensures that our main conclusions are not driven by the range difference. See Supplementary Figures 10 and 11. All tests refer to two-sided hypotheses and were calculated with the lmerTest package in R [19]. P-values are not adjusted for multiple comparisons.

Supplementary References

[1] Mannix E, Neale MA (2005) What Differences Make a Difference? *Psychological Science in the Public Interest* 6(2):31–55.

[2] Phillips KW, Loyd DL (2006) When surface and deep-level diversity collide: The effects on dissenting group members. *Organizational Behavior and Human Decision Processes* 99(2):143–160.

[3] van Dijk H, Meyer B, van Engen M, Loyd DL (2017) Microdynamics in Diverse Teams: A Review and Integration of the Diversity and Stereotyping Literatures. *Academy of Management Annals* 11(1):517–557.

[4] AlShebli BK, Rahwan T, Woon WL (2018) The preeminence of ethnic diversity in scientific collaboration. *Nature Communications* 9(1):5163.

[5] Page SE (2007) *The Difference How the Power of Diversity Creates Better Groups, Firms, Schools, and Societies.* (Princeton University Press).

[6] Woolley AW, Chabris CF, Pentland A, Hashmi N, Malone TW (2010) Evidence for a collective intelligence factor in the performance of human groups. *Science (New York, N.Y.)* 330(6004):686–8.

[7] Frederick S (2005) Cognitive Reflection and Decision Making. *Journal of Economic Perspectives* 19(4):25–42.

[8] Stanovich KE, West RF (1998) Individual differences in rational thought. *Journal of Experimental Psychology: General* 127(2):161–188.

[9] Cacioppo JT, Petty RE, Kao CF (1984) The Efficient Assess- ment of Need for Cognition. *Journal of Personality Assessment* 48(3):306 – 07.

[10] Ester M, Kriegel HP, Sander J, Xu X (1996) A density-based algorithm for discovering clusters in large spatial databases with noise in *Proceedings of the Second International Conference on Knowledge Discovery and Data Mining (KDD-96).* (AAAI Press), pp. 226–231.

[11] Navajas J, Niella T, Garbulsky G, Bahrami B, Sigman M (2018) Aggregated knowledge from a small number of debates outperforms the wisdom of large crowds. *Nature Human Behaviour* 2(2):126–132.

[12] Brier GW (1950) Verification of Forecasts Expressed in Terms of Probability. *Monthly Weather Review* 78(1):1–3.

[13] Fleming SM, et al. (2014) Action-Specific Disruption of Perceptual Confidence. *Psychological science.*

[14] Lo S, Andrews S (2015) To transform or not to transform: using generalized linear mixed models to analyse reaction time data. *Frontiers in Psychology* 6.

[15] Fechner GT (1860) *Elemente der Psychophysik [Elements of psychophysics].* (Breitkopf und Härtel., Leipzig).

[16] Kao AB, et al. (2018) Counteracting estimation bias and social influence to improve the wisdom of crowds. *Journal of The Royal Society Interface* 15(141):20180130.

[17] Tetlock PE (2006) *Expert Political Judgment: How Good Is It? How Can We Know?* (Princeton University Press, Princeton).

[18] Hofmann H, Wickham H, Kafadar K (2017) Letter-Value Plots: Boxplots for Large Data. *Journal of Computational and Graphical Statistics* 26(3):469–477.

[19] Kuznetsova A, Brockhoff PB, Christensen RHB (2017) lmerTest Package: Tests in Linear Mixed Effects Models. *Journal of Statistical Software* 82(13).

[20] Biemann T, Kearney E (2010) Size Does Matter: How Varying Group Sizes in a Sample Affect the Most Common Measures of Group Diversity. *Organizational Research Methods* 13(3):582–599.
