# Supplementary material for: Modularity and composite diversity affect the collective gathering of information online
Source: Nat Commun. 2021 May 27;12:3195. doi: 10.1038/s41467-021-23424-1 (PMC8159948; doi:10.1038/s41467-021-23424-1)
Supplement: Supplementary file 3 — Reporting Summary [file 41467_2021_23424_MOESM3_ESM.pdf]

nature research

Corresponding author(s): Niccolo Pescetelli

Last updated by author(s): Jan 1, 2021

Reporting Summary

Nature Research wishes to improve the reproducibility of the work that we publish. This form provides structure for consistency and transparency in reporting. For further information on Nature Research policies, see Authors & Referees and the Editorial Policy Checklist.

Statistics

For all statistical analyses, confirm that the following items are present in the figure legend, table legend, main text, or Methods section.

n/a	Confirmed	
☐	☒	The exact sample size (n) for each experimental group/condition, given as a discrete number and unit of measurement
☐	☒	A statement on whether measurements were taken from distinct samples or whether the same sample was measured repeatedly
☐	☒	The statistical test(s) used AND whether they are one- or two-sided<br>*Only common tests should be described solely by name; describe more complex techniques in the Methods section.*
☐	☒	A description of all covariates tested
☐	☒	A description of any assumptions or corrections, such as tests of normality and adjustment for multiple comparisons
☐	☒	A full description of the statistical parameters including central tendency (e.g. means) or other basic estimates (e.g. regression coefficient) AND variation (e.g. standard deviation) or associated estimates of uncertainty (e.g. confidence intervals)
☐	☒	For null hypothesis testing, the test statistic (e.g. F, t, r) with confidence intervals, effect sizes, degrees of freedom and P value noted<br>*Give P values as exact values whenever suitable.*
☒	☐	For Bayesian analysis, information on the choice of priors and Markov chain Monte Carlo settings
☐	☒	For hierarchical and complex designs, identification of the appropriate level for tests and full reporting of outcomes
☐	☒	Estimates of effect sizes (e.g. Cohen's d, Pearson's r), indicating how they were calculated

Our web collection on statistics for biologists contains articles on many of the points above.

Software and code

Policy information about availability of computer code

Data collection	Data was collected online with a custom web app made freely available.
Data analysis	Analysis was performed in Python 3.5 and R v3.6.3. Code is available via OSF: https://osf.io/wb538

For manuscripts utilizing custom algorithms or software that are central to the research but not yet described in published literature, software must be made available to editors/reviewers. We strongly encourage code deposition in a community repository (e.g. GitHub). See the Nature Research guidelines for submitting code & software for further information.

Data

Policy information about availability of data

All manuscripts must include a data availability statement. This statement should provide the following information, where applicable:

- Accession codes, unique identifiers, or web links for publicly available datasets
- A list of figures that have associated raw data
- A description of any restrictions on data availability

data to reproduce figures and analysis is available via OSF: https://osf.io/wb538

Field-specific reporting

Please select the one below that is the best fit for your research. If you are not sure, read the appropriate sections before making your selection.

☐ Life sciences ☒ Behavioural & social sciences ☐ Ecological, evolutionary & environmental sciences

For a reference copy of the document with all sections, see nature.com/documents/nr-reporting-summary-flat.pdf

Behavioural & social sciences study design

All studies must disclose on these points even when the disclosure is negative.

Study description	The behavioral study asked online groups of volunteers to answer forecasting questions, find task relevant information online and discuss it with others. The purpose of the study was to understand how collective representations of geopolitical events are shaped by social and technological factors, such as search engines used to find information, online groups composition and size.
Research sample	Volunteers were recruited on Amazon Mechanical Turk and represent representative population of United States. 193 people completed the initial questionnaire. 109 people returned on the day of the main experiment (mean age = 18, 45 females). Given the nature of the subject (ie. online collaboration) we opted for recruitment via Amazon Mechanical Turk, a standard recruitment platform for studies in collective intelligence and computational social social science. This also ensured that volunteers were likely to be digitally savvy.
Sampling strategy	Participants were assigned to one of four experimental conditions (low diversity small group, low diversity large group, high diversity small group, high diversity large group). Given the nature of the experimental procedure (group experiment), sample sizes depended on the number of volunteers who responded to the study ad in the recruitment period (3 days before the test) and returned 3 days after for the main test. Only the former was under the experimenter's control. We set out to screen at pre-test at least 150 volunteers (corresponding to about 35 people in each condition). Our calculations were informed by previous literature on group experiments, although no formal power analysis was performed.
Data collection	All participants were recruited and tested online via a custom developed web application developed with Heroku. The data was collected on a database connected to the application and downloaded soon after the experiment was ran. The experimenter was not blind to the experimental condition or hypothesis but did not interact with the participants during data collection.
Timing	Test data was collected 23 March 2018. Pre-test questionnaires were collected 3 days prior.
Data exclusions	2 groups (5 people in total) had to be excluded from the analysis due to the fact that the manipulation procedure did not work due to dropout. This is also reported in the manuscript, in supplementary information (Table S1-S2).
Non-participation	193 completed the pre-test phase. 104 completed the test phase.
Randomization	Participants in the Core segment of our sample were randomized in the four conditions. Participants in the Inner and Outer segment where randomized only to the size condition.

Reporting for specific materials, systems and methods

We require information from authors about some types of materials, experimental systems and methods used in many studies. Here, indicate whether each material, system or method listed is relevant to your study. If you are not sure if a list item applies to your research, read the appropriate section before selecting a response.

Materials & experimental systems

n/a	Involved in the study	
☒	☐	Antibodies
☒	☐	Eukaryotic cell lines
☒	☐	Palaeontology
☒	☐	Animals and other organisms
☐	☒	Human research participants
☒	☐	Clinical data

Methods

n/a	Involved in the study	
☒	☐	ChIP-seq
☒	☐	Flow cytometry
☒	☐	MRI-based neuroimaging

Human research participants

Policy information about studies involving human research participants

Population characteristics	see above
Recruitment	Recruitment was carried out online via Amazon Mechanical Turk. Although highly representative of western English speaking population, this procedure might be biased toward digitally savvy individuals. Speculatively, the effects described in the study may be less pronounced in less digitally savvy individuals.
Ethics oversight	MIT IRB board

Note that full information on the approval of the study protocol must also be provided in the manuscript.
